# Biosynthesis of ecofriendly antibacterial nanoparticles with healing effects in a murine diabetic skin infection model

**DOI:** 10.1038/s41598-026-54908-z

**Published:** 2026-06-15

**Authors:** Eman A. Mustafa, Hanady G. Nada, Walaa A. Eraqi, Rehab N. Shamma, Hala N. Elhifnawi, Nourtan F. Abdeltawab

**Affiliations:** 1 Postgraduate Program in Microbiology and Immunology, Faculty of Pharmacy, Cairo University, Cairo, Egypt; 2 Drug Radiation Research Department, National Center for Radiation Research and Technology, Egyptian Atomic Energy Authority, Cairo, Egypt; 3 Department of Microbiology and Immunology, Faculty of Pharmacy, Cairo University, Cairo, 11562 Egypt; 4 Department of Pharmaceutics and Industrial Pharmacy, Faculty of Pharmacy, Cairo University, Cairo, 11562 Egypt

**Keywords:** Diabetes mellitus, Green tea, Gamma radiation, Antibacterial activity, Diabetic skin infections, Biotechnology, Drug discovery, Microbiology

## Abstract

Diabetes mellitus is a global concern with complications including recurrent skin infections and poor wound healing. The involvement of multidrug-resistant bacteria (MDR) in skin infections renders them challenging. Nanoparticles such as silver, zinc oxide, and chitosan-based nanoparticles offer a promise for combating the growing threat of MDR bacteria by employing unique mechanisms that bypass recognized antibiotic resistance pathways. Therefore, this study reports the eco-friendly synthesis of silver (AgNPs), zinc oxide (ZnONPs), and chitosan-tripolyphosphate nanoparticles (Cs-TPP-NPs) using green tea extract (GTE) and gamma irradiation. AgNPs exhibited the highest antibacterial and antibiofilm efficacy among the tested preparations, as reflected by their lowest minimum inhibitory concentration (MIC) and minimum bactericidal concentration (MBC) values against four bacterial strains associated with diabetic foot ulcers, including *Pseudomonas aeruginosa*, *Escherichia coli*, methicillin-resistant *Staphylococcus aureus* (MRSA), and *Streptococcus pyogenes*. The killing activity of AgNPs was fast acting against all tested bacteria with > 10 log_10_ CFU/mL reduction in 2–6 h. The AgNPs-GTE loaded Cs-TPP-NPs combination was assessed for possible mechanisms of action, which revealed its ability to inhibit biofilm formation and compromise bacterial membrane integrity in MRSA and *E. coli*, leading to increased permeability, intracellular ion leakage, and nucleic acid release. Notable morphological damage to bacterial cells was confirmed by transmission electron microscope (TEM).  In vivo, the prepared combination hydrogel promoted healing of skin lesions in a CD1  mouse model of diabetic skin infection lesion within six days, demonstrating strong antibacterial activity with a 4 Log_10_ unit reduction in bacterial load compared with the untreated group, and significant decrease in inflammatory marker NF-kB, restoring it to healthy uninfected control levels. Histopathological evaluation of treated tissues showed full epidermal regeneration, enhanced fibroblast proliferation, increased angiogenesis, and the presence of well-organized mature collagen fibers in the dermis, compared to untreated controls. In conclusion, synthesized AgNPs-GTE loaded Cs-TPP-NPs combination offered an effective therapeutic approach for enhancing diabetic skin infection healing.

**Supplementary Information:**

The online version contains supplementary material available at 10.1038/s41598-026-54908-z.

## Introduction

Diabetes mellitus (DM) is a metabolic disorder characterized by impaired insulin secretion or function, leading to hyperglycemia^1^. According to the World Health Organization (WHO), it was the eighth leading cause of death in 2019, accounting for approximately 1.6 million deaths worldwide^2^. DM is increasingly recognized as a chronic inflammatory disorder. Patients with diabetes exhibit significantly elevated levels of pro-inflammatory cytokines, including Interleukin-6 (IL-6), Interleukin-18 (IL-18), Interleukin-1 (IL-1), and tumor necrosis factor-alpha (TNF-α), along with increased expression of inflammatory mediators such as intercellular adhesion molecule-1 (ICAM-1), vascular cell adhesion molecule-1 (VCAM-1), and nuclear factor-κB (NF-κB)^3^. Diabetic foot infection (DFI) is a severe complication of diabetes associated with high morbidity and increased healthcare burden^4^. It is usually caused by infection or deep tissue damage, often accompanied by distal neuropathy and varying degrees of peripheral vascular disease in the lower extremities. Approximately 15% of patients with diabetes are at risk of developing diabetic foot infection in their lifetime^5^. Management of the infectious diabetic foot remains a global health challenge. Effective infection control is essential to prevent amputation and reduce healthcare costs^6^.

Chronic diabetic foot infections are often polymicrobial and commonly involve both Gram-positive and Gram-negative bacteria, including *Staphylococcus aureus*, *Pseudomonas aeruginosa*, *Escherichia coli,* and *Streptococcus pyogenes,* as well as other species such as *Enterococcus* and *Proteus mirabilis*, in addition to anaerobic pathogens^5^. *Staphylococcus aureus* is the most frequently isolated microbe in diabetic foot infections^7,8^, with methicillin-resistant *S. aureus* (MRSA) reported in approximately 15–30% of cases^9^. To model these commonly encountered pathogens, the present work used four well-characterized reference strains of MRSA N315, *E. coli* K12 MG1655, *P. aeruginosa* PAO1, and *S. pyogenes* ATCC 19615.

Antimicrobial resistance (AMR), particularly among multidrug-resistant (MDR) pathogens listed by the World Health Organization as priority organisms, is a significant global health concern that compromises the effectiveness of many commonly used antibiotics^10^. These pathogens are responsible for a considerable percentage of hospital-acquired infections and demonstrate resistance to numerous first-line therapies^11^. A variety of factors, including prolonged wound healing, multiple hospital admissions, inadequate antibiotic therapy and peripheral artery disease, may increase the prevalence of multidrug-resistant microbes in people with diabetic skin infections^12^. To overcome the antibiotic resistance problem, there is a pressing need to explore alternative and innovative therapies using natural plant extracts^4^ either separately or in combination with antibiotics, probiotics, natural and modified proteins, nanoparticles, and phage therapy^13^.

Medicinal plants have been used for their diverse bioactive compounds, including polyphenols, flavonoids, tannins, saponins and alkaloids, which exhibit a range of biological activities such as antimicrobial, antioxidant and anti-inflammatory effects^14^. Some traditional herbal medicines, such as *Astragali Radix*, have been reported to show antidiabetic effects by modulating gut microbiota, reducing insulin resistance, and lowering blood glucose levels^15^, along with others like *Balanites aegyptiaca*, *Nigella sativa*, and *Aloe vera*^14^. Green tea (*Camellia sinensis*) is well known for its high polyphenol content. Epigallocatechin-3- gallate (EGCG) is a natural anti-inflammatory compound considered the most common polyphenol in green tea leaves with excellent bioactivities, such as antioxidant, free radical scavenging, anti-inflammatory and antibacterial effects^16,17^. Moreover, green tea catechins exhibit a wide antibacterial activity against Gram-positive and Gram-negative bacteria through different mechanisms, such as, the inhibition of synthesis of cell wall, cell membrane, protein and nucleic acid, or the inhibition of metabolic pathways, including those involving toxins, extracellular matrix virulence factors, and oxidative stress^18,19^. The inflammatory phase, occurring within the first 48 h after skin injury, is crucial for clearing pathogens and preparing the wound for healing^20^. Immune cells like neutrophils, monocytes, and macrophages migrate to the infection site and release signaling molecules. However, if not controlled, substances like proteases and reactive oxygen species (ROS) from neutrophils can cause tissue damage^21^. EGCG in green tea has been shown to reduce neutrophil infiltration, as well as monocyte migration and adherence^16^. Furthermore, green tea leaves are characterized by the presence of high content of various phytochemical constituents that act as reducing and stabilizing agents of metallic ions into nanoparticles^22^.

Over the last two decades, the use of nanomaterials (NMs) has greatly expanded^23^. Studies have demonstrated that several pharmaceutical nanoparticles are highly effective in accelerating skin regeneration, as well as infection prevention and antibacterial activity against MDR strains^24^. The ability of metal nanoparticles to target several bacterial metabolic processes makes them an attractive approach for managing antibiotic resistance in skin infections. Therefore, the combination of metals with other nanomaterials and polymers presents a promising strategy for developing effective wound dressings^25^. Among these metals, silver nanoparticles (AgNPs) have demonstrated extraordinary wound healing efficacy. AgNPs can disrupt bacterial cell membranes via cytotoxicity, resulting in bacterial cell death. AgNPs function as both antibacterial agents and wound healing accelerators. Furthermore, AgNPs have a mediating function in wound healing because of their anti-inflammatory activity^1^. Zinc oxide nanoparticles (ZnONPs) are one of the most important antimicrobial agents that have recently attracted significant interest. They have broad-spectrum antimicrobial activity against many pathogenic microbial strains^26^. ZnONPs have been recognized for their important contributions to wound healing, marked by their antibacterial activity, sustained zinc release, and ability to stimulate collagen breakdown in necrotic tissue, aiding tissue cleanup and wound healing^27^. Chitosan, a natural polymer with antibacterial, biocompatibility, and adhesive properties, is a very effective material for the development of hydrogels, which have great potential for use in the treatment of diabetic skin infections^17^. Chitosan is positively charged and by itself can kill bacteria by interacting with the negative charge on the surface of the bacterial cell membrane^28^.

This study aimed to evaluate the antimicrobial potency of irradiated green tea extract and multiple ecofriendly nanoparticles against pathogenic bacteria associated with diabetic skin infections. This work also examined the use of gamma-irradiated green tea extract as a basis for formulating a hydrogel that incorporates eco-friendly nanoparticles and chitosan. This combined system was designed to enhance healing, antibacterial, and anti-inflammatory activity in a murine diabetic skin infection lesion model. By integrating a naturally derived, gamma-decontaminated extract with biocompatible nanomaterials, the study assessed a potential strategy for managing infected diabetic wounds while reducing reliance on conventional antibiotics.

## Methods

### Bacterial strains, culturing conditions, and maintenance

Four bacterial strains were used in this study, representing key Gram-positive and Gram-negative pathogens associated with diabetic skin infections. These strains included methicillin-resistant *Staphylococcus aureus* N315^29^, *Escherichia coli* K12MG1655^30^, *Pseudomonas aeruginosa* PAO1^31^, and *Streptococcus pyogenes* ATCC 19615^32^. The identity and purity of all strains were authenticated in-house upon receipt and prior to experimental use through Gram staining, observation of characteristic colony morphology on selective media, and biochemical profiling. Furthermore, the identity of the *E. coli* K12MG1655 strain was confirmed via Whole Genome Sequencing (WGS) to verify genomic identity. *S. aureus* N315 and *P. aeruginosa* PAO1 were cultured on tryptic soy agar (TSA), *E. coli* K12MG1655 on MacConkey agar, and *S. pyogenes* ATCC 19615 on blood agar. Following incubation at 37°C for 24 h, a pure colony was sub-cultured on tryptic soy broth (TSB) for stock preparation. Microbial stocks were preserved in TSB supplemented with 25% (v/v) glycerol and maintained at—80 ˚C freezer (Thermo Fisher Scientific, Waltham, MA, USA).

### Decontamination of green tea by gamma radiation

Gamma (γ)- irradiation was used as a decontamination method for green tea. GT samples (1g each) were packed in screw capped test tubes and exposed to γ – radiation at four different doses (1, 3, 5 and 7 kGy) using Cobalt-60 220 gamma cell, Canada Co. Ltd, located at the National Center for Radiation Research and Technology (NCRRT), Cairo, Egypt. The radiation rate was 1 kGy/h at the time of the experiment^33^.

### Testing the effect of γ – radiation on the microbial load and test for pathogens

One gram of each irradiated sample, along with a non-irradiated sample as a negative control, was suspended in 9 ml of sterile saline solution, mixed vigorously, and serially diluted for microbial enumeration. The bacterial count was determined using standard plate count method on nutrient agar (NA) medium (Oxoid, Hampshire, UK) at 37°C incubated for 24–48 h, while the fungi were counted on sabouraud dextrose agar medium (SDA) (Oxoid, Hampshire, UK) medium and incubated at 25°C for 3–5 days. The count was determined and expressed as CFU/ml^34^, which represents the number of viable bacterial cells capable of forming colonies in one milliliter of the sample. Samples that presented bacterial growth greater than 10^5^ CFU/ml were considered unsatisfactory according to WHO guidelines for aerobic bacteria. The most effective radiation dose was chosen for the next experiment.

To test for pathogens following US pharmacopeia^35^, ten grams of samples of irradiated and non-irradiated green tea (GT) (Ahmed tea, London, England) were first dispersed in 100 ml of TSB (Oxoid, Hampshire, UK) and incubated at 37°C for 2 h in preparation for bacterial pathogens detection. Similarly, in preparation for select fungal pathogen detection, 10 g of irradiated and non-irradiated GT were dispersed in 100 ml of sabouraud dextrose broth (SDB) (Oxoid, Hampshire, UK) and incubated at 25°C for 24 h. Bacterial pathogens detection involved sub-culturing 0.1 ml of samples in TSB media on MacConkey (Oxoid, Hampshire, UK) and eosin methylene blue (EMB) (Oxoid, Hampshire, UK) for detection of *E. coli*; mannitol salt agar (MSA) (Oxoid, Hampshire, UK) for detection of *S. aureus*; cetrimide agar (Oxoid, Hampshire, UK) for detection of *P. aeruginosa*; and salmonella-shigella agar (SSA) (Oxoid, Hampshire, UK) for detection of *salmonella* species, followed by incubation at 37°C for 48 h. *Candida albicans* was detected by sub-culturing on SDA at 25°C for 3–5 days. Characteristic colony formations confirmed the presence of each microorganism. All the experiments were performed in triplicates and repeated on three independent times.

### Analysis of green tea components integrity before and after exposure to γ – radiation by HPLC

High-performance liquid chromatography (HPLC) was used as an external standard method to evaluate the effect of the selected dose of gamma irradiation on GT extract components. The freeze-dried extract, before and after gamma irradiation were analyzed using HPLC to determine the amount of catechins components by comparing chromatograms of the samples before and after gamma irradiation with the epigallocatechin gallate standard (EGCG) (Nawah Scientific, Mokattam, Egypt) chromatogram. The mobile phase consisted of acetonitrile and glacial acetic acid. An ultrasonic bath was used to remove dissolved gases. Serial dilutions of EGCG standard solution were prepared for comparison. Detection was carried out using a UV–visible detector at 280 nm, with flow rate of 1 ml/min and an injection volume of 10 µl. The peak area described the quantity of bioactive compound in the solution^36^. An HPLC analysis was performed using an Agilent HPLC 1200 system (Agilent Technologies, Palo Alto, CA, USA) equipped with a photodiode array detector (PDA; 1260 Infinity series, Agilent Technologies).

### Silver and zinc oxide nanoparticles synthesis

Silver nanoparticles (AgNPs) were synthesized as described by Rónavári et al.^37^. A 0.1 M AgNO_3_ aqueous solution was prepared by dissolving AgNO_3_ (Sigma- Aldrich, Germany) in 100 ml deionized water. At the same time, 2 g of dry GT leaves’ powder was soaked in 100 ml distilled water and heated to 80°C for 20 min. Zinc oxide nanoparticles (ZnONPs) were synthesized as described by Dhanemozhi et al.^38^ using a 0.1M zinc acetate dihydrate (Sigma- Aldrich, Germany) solution in 60 ml of deionized water with stirring for a few minutes using a magnetic stirrer (IKA® C-MAG HS, Staufen, Germany). Simultaneously, 5 g of GT leaves’ powder were dissolved in 100 ml of distilled water and magnetically stirred for 1 h at 70°C. Both prepared green tea infusions (GTI) were left to cool to room temperature and filtered through Whatman No. 1 filter paper, followed by a 25-μm filter. The prepared green tea filtrates were mixed homogenously with the prepared salt solutions in a 1:1 volume ratio for AgNPs and 1:1.5 volume ratio for ZnONPs, then continuously stirred for 24 h. The GT filtrate acted as both a reducing agent and a stabilizer of the synthesized NPs. The mixtures were exposed to 7 kGy gamma irradiation to enhance reduction and stabilization of the synthesized NPs. Finally, the prepared NPs were purified by repeated ultracentrifugation at 20.000 rpm for 10 min, followed by washing with deionized water to remove any surface adsorptions, then reseparated by ultracentrifugation^39^.

### Synthesis of green tea extract loaded chitosan tri-poly phosphate nanoparticles

GTE was prepared by boiling 10 g of dried green tea leaves’ powder in 100 ml distilled water for 10 min. The extract was then filtered through Whatman No. 1 filter paper and lyophilized^40^. GTE-loaded chitosan-tripoly phosphate nanoparticles (GTE loaded CS-TPP-NPs) were then prepared by ionic gelation method^41^. Briefly, chitosan (Cs) powder (Sigma- Aldrich, Germany) was dissolved in 8 ml of 1% (v/v) acetic acid solution (pH 3) to achieve a final formula composition of 0.25%. The cross-linker phase was composed of 2 ml 0.0625% of TPP solution, yielding a Cs: TPP volume ratio of 4:1. In this phase, 10 mg of the lyophilized GTE were dissolved using water bath sonicator (FB15051, Fisher Scientific, Waltham, MA, USA). The mixture was then added dropwise to the CS solution under magnetic stirrer until a translucent nanoparticle suspension was formed, followed by stirring for 1 h at 1000 rpm at ambient temperature.

### Characterization of different nanoparticles

Various analytical techniques were used for the characterization of the prepared nanoparticles to determine their size, functional groups, shape, and crystallographic structure. The optical properties of biosynthesized AgNPs and ZnONPs were studied using UV–Visible spectrophotometer (JASCO V-560. UV–Vis. Spectrophotometer) by scanning in the wavelength range 200–900 nm at a resolution of 1.0 nm using the green tea filtrate as a negative control for autozero support^42^. Additionally, Fourier Transform Infrared Spectroscopy (FTIR) were obtained using an IR-spectrophotometer (JASCO FTIR 3600 Infrared spectrometer, Japan) providing information regarding chemical functional groups in the GTE. FTIR was recorded at a resolution of 4.0cm^-1^ in a wave range of 400- 4000 cm^-1^ using the KBr Pellet method^43^. The size and morphology of the nanoparticles were analyzed using a Transmission Electron Microscope (TEM; JEOL JEM-100 CX). Before imaging, the NP samples were dried in an autoclave at 37.0 ± 2 °C and drop-coated onto carbon-coated TEM grids^42^.

To determine the average particle size, size distribution and surface charge of the synthesized nanoparticles, dynamic light scattering (DLS) and zeta potential was performed using a PSS-NICOMP™ 380 ZLS system (St. Barbara, California, USA)^44^. Moreover, X-ray diffraction (XRD) analysis was performed to determine the crystallinity, crystallite size, and lattice structure of the produced nanoparticles using an XRD-6000 diffractometer (Shimadzu, SSI, Japan). The diffraction angle (2θ) was recorded to measure the intensity of the diffracted X-rays. Calcination of ZnONPs at 500° C for 2 h was performed to enhance crystallinity of the synthesized ZnONPs^45^.

The elemental composition, purity, and relative proportions of the elements present in the samples were analyzed using Energy Dispersive X-ray Spectroscopy (EDS). Additionally, elemental mapping was performed following SEM/EDS analysis (JEOL JSM-5600 LV, Japan) to provide detailed information on the purity, distribution, and localization of the detected elements within the synthesized nanoparticles^43^.

### Screening of antimicrobial activity of different preparations

As a preliminary step, the antibacterial activity of the preparations was evaluated using the agar well-diffusion method^46^. Six preparations were assessed for their antimicrobial activity, namely AgNPs, ZnONPs, GTE loaded Cs-TPP-NPs, EGCG, GTE and GTI. Ciprofloxacin was included as a positive control. The activity of these preparations was examined against the four standard bacterial strains commonly associated with diabetic foot ulcers namely *P. aeruginosa* PAO1,  *E. coli* K12MG1655, methicillin resistant *S. aureus* N315, and *S. pyogenes ATCC 19615*.

Bacterial suspensions of each standard strain were prepared and adjusted to an optical density of 0.08 at 600 nm (OD_600_). The adjusted bacterial suspensions were uniformly swabbed on Mueller–Hinton agar (MHA) (Oxoid, Hampshire, UK) plates. Wells (6 mm in diameter) were made using a sterile cork-borer and filled with 50 µl of the different preparations. The plates were incubated at 37° C for 24 h, and the diameters of the inhibition zone was measured in mm to evaluate the antibacterial activity. All assays were performed in triplicates and repeated on three independent times.

### Determination of minimum inhibitory concentration and minimum bactericidal concentration of different preparations

The minimum inhibitory concentrations (MIC) of the preparations (AgNPs, ZnONPs, GTE loaded Cs-TPP-NPs, EGCG, GTE and GTI) in comparison with ciprofloxacin as a positive control, were determined according to the guidelines of the Clinical and Laboratory Standards Institute (CLSI) using the broth microdilution method^47^ . In sterile 96-well plates, 140 μl of Mueller–Hinton (MHB) (Oxoid, Hampshire, UK) and 10 μl of the adjusted bacterial suspensions as described before were added. TSB was used instead of MHB for *S. pyogenes*. Blank wells contained only media, while the negative control wells contained bacterial culture and broth. The test wells contained bacterial culture and 50 μl of tested preparations at different concentrations from 100% to 0.19% (v/v). The plates were incubated at 37 °C for 24 h in a stationary condition. Subsequently, the MIC value for each of the preparations was recorded as the lowest concentration showing no visible growth.

To determine the minimum bactericidal concentration (MBC) values, a 5 μl of each well showing no visible growth were spread on TSA plates and incubated for 24–48 h at 37°C. MBCs of the each preparations was determined as the lowest concentration at which no colony formation occurred^47^ .

The MBC/MIC ratio was used to define the mode of activity of different preparations: bactericidal when scores are 1, 2 and 4 or bacteriostatic if score > 4^48^. The experiment was performed in triplicates and repeated on three independent times.

### Effect of the different preparations on biofilm formation

A quantitative evaluation of the anti-biofilm activity of the preparations (AgNPs, ZnONPs, GTE loaded Cs-TPP-NPs, EGCG, GTE and GTI) in comparison with ciprofloxacin as a positive control, was performed using a crystal violet microtiter plate assay^49^ . Briefly, 10 μl of the adjusted bacterial suspensions as previously described was added to each well of a 96-well flat-bottom microtiter plate containing 140 μl of TSB. Three wells served as a medium control (blank), control well contained only bacterial culture and broth. In the test wells, 50 μl of different preparations were added to sub- MIC concentrations (1/2 MIC) and incubated at 37°C for 24 h. The next day, the contents of the wells were discarded, and wells were gently washed with sterile phosphate buffer saline (PBS) (Lonza, Basel, Switzerland), then discarded and left to dry for 30 min. Subsequently, the wells were stained with 200 μl crystal violet (0.1% w/v) (British Drug Houses Ltd. London, England) and incubated for 15 min at room temperature. Following incubation, wells were washed with distilled water several times and left to dry. Subsequently, 200 μl of 70% ethanol (El Nasr Pharmaceutical Chemicals Co. Adwic, Cairo, Egypt) was added to each well. The optical density was measured at 630 nm using a Multiskan Sky Microplate Spectrophotometer. The experiment was performed in triplicates and repeated on three independent times.

### Determination of time-kill kinetics of different preparations

The time–kill kinetics of different preparations including, (AgNPs, ZnONPs, GTE loaded Cs-TPP-NPs, EGCG, GTE and GTI) in comparison with ciprofloxacin as a positive control, were evaluated against four standard bacterial strains: *P. aeruginosa*, *E. coli*, MRSA and *S. pyogenes*. Each preparation was tested at its MBC concentrations and incubated with bacterial cultures adjusted to 0.08 at OD_600_. The bacterial culture without any treatment was used as a positive control. The killing capacity at 0, 2, 4, 8, 10, 12, 18 and 24 h, were assessed using broth micro-dilution method. At each time point, 10 μl of each well as serially diluted 10-folds and plated on TSA plates to determine the viable colony count^50^. The experiment was performed in triplicates and repeated on three independent times.

### Determination of fractional inhibitory concentration index for different combinations

The antibacterial activity of GTE and three synthesized nanoparticles (AgNPs, ZnONPs and GTE loaded Cs-TPP-NPs) was evaluated individually and in 1:1 combinations using the broth microdilution assay^51^ . Two-fold serial dilutions of each preparation and combination, ranging from 100% to 0.19% (v/v), were prepared. In a sterile 96-well plate, 50 μl of each dilution was mixed with 140 μl of sterile TSB. A 10 μl bacterial suspension, adjusted to an OD₆₀₀ of 0.08, was then added to each well. The plates were incubated at 37°C for 24 h then MIC and MBC value for each combination were determined. The MIC was identified as the lowest concentration in the well where no visible bacterial growth was observed. The experiment was performed in triplicates and repeated on three independent times. The values were recorded, and the fractional inhibitory concentration index (ΣFIC) was calculated as follow:

$$\mathrm{F}\mathrm{I}\mathrm{C}(\mathrm{A})=\frac{\mathrm{M}\mathrm{I}\mathrm{C} \,\,\mathrm{o}\mathrm{f} \,\,(\mathrm{a}*) \,\,\mathrm{c}\mathrm{o}\mathrm{m}\mathrm{b}\mathrm{i}\mathrm{n}\mathrm{e}\mathrm{d}\,\, \mathrm{w}\mathrm{i}\mathrm{t}\mathrm{h} \,\,(\mathrm{b}*)}{\mathrm{M}\mathrm{I}\mathrm{C} \,\,\mathrm{o}\mathrm{f} \,\,(\mathrm{a}) \,\,\mathrm{i}\mathrm{n}\mathrm{d}\mathrm{e}\mathrm{p}\mathrm{e}\mathrm{n}\mathrm{d}\mathrm{e}\mathrm{n}\mathrm{t}\mathrm{l}\mathrm{y}}$$

$$\mathrm{F}\mathrm{I}\mathrm{C}(\mathrm{B})=\frac{\mathrm{M}\mathrm{I}\mathrm{C} \,\,\mathrm{o}\mathrm{f} \,\,(\mathrm{b}*) \,\,\mathrm{c}\mathrm{o}\mathrm{m}\mathrm{b}\mathrm{i}\mathrm{n}\mathrm{e}\mathrm{d}\,\, \mathrm{w}\mathrm{i}\mathrm{t}\mathrm{h} \,\,(\mathrm{a}*)}{\mathrm{M}\mathrm{I}\mathrm{C}\,\, \mathrm{o}\mathrm{f} \,\,(\mathrm{b}) \,\,\mathrm{i}\mathrm{n}\mathrm{d}\mathrm{e}\mathrm{p}\mathrm{e}\mathrm{n}\mathrm{d}\mathrm{e}\mathrm{n}\mathrm{t}\mathrm{l}\mathrm{y}}$$

*Where (a*) and (b*) are components in the combination.

From these values, the FIC index was calculated accordingly:

$$\Sigma {\text{FIC }} = {\text{ FIC }}\left( {\mathrm{A}} \right) \, + {\text{ FIC }}\left( {\mathrm{B}} \right)$$

The ΣFIC was interpreted as ≤ 0.5 indicating synergy; > 0.5 and ≤ 1.0 as additive; > 1.0 and ≤ 4.0 indicating indifference; and > 4.0 indicating antagonism.

### Assessment of possible mechanisms of action of the selected combination against  *E. coli* and MRSA

The most effective combination (AgNPs-GTE loaded Cs-TPP-NPs) was assayed for its possible antibacterial mechanism of action using multiple assays, including induced morphological alterations in the tested bacteria, loss of membrane integrity and leakage of intracellular components.

### Visualization of the effect of the selected combination on bacterial cells by transmission electron microscope

Cellular morphological alterations of bacteria treated with AgNPs-GTE loaded Cs-TPP-NPs was observed by transmission electron microscopy (TEM). Bacterial suspensions of *E. coli* and MRSA were prepared, their OD_600_ nm was adjusted to 1 then the MBC of the selected combination was added to each suspension and incubated for 6 h. The bacterial suspensions were then centrifuged and washed twice using a PBS and treated with 2.5% glutaraldehyde overnight. The samples were prepared according to a previously published protocol^52^ and observed by TEM photography using JEOL JEM-1400 Flash electron microscope 120 kV, with a resolution of 0.2 nm and magnification power of 10 to 1.200.000, (JEOL Ltd., Tokyo, Japan) at the Transmission Electron Microscope (TEM) Unit, Faculty of Agriculture, Cairo University, Cairo, Egypt.

### Visualization of the effect of the selected combination on biofilm by light microscopy

The effect of the selected combination (AgNPs-GTE loaded Cs-TPP-NPs) at its sub-MIC concentration (1/2 MIC) was tested against biofilm formation by *E. coli* and MRSA on glass surfaces according to a previously published protocol^53^. For microscopic visualization, the biofilm was allowed to grow on glass slides in the absence and presence of tested combination as described in the crystal violet assay. Following incubation, the slides were gently washed with PBS to remove non-adherent cells, and the remaining biofilm was stained with 0.1% (w/v) crystal violet. Excess stain was removed by washing with distilled water, and the slides were air-dried. The stained biofilms were then examined under a bright-field microscope (Euromex iScope series 110–240 V / 50–60 Hz) at 40 × magnification and imaged using a digital camera attached to the microscope.

### Visualization of the effect of the selected combination on biofilm by scanning electron microscopy

*E. coli* and MRSA suspensions were incubated overnight in TSB at 37°C, then adjusted to an optical density of 0.5 at 600 nm. Then treated and untreated suspensions allowed to form biofilm on sterile glass coverslips to obtain samples for scanning electron microscopy (SEM)^54^. After fixation with 2.5% glutaraldehyde solution for 2 h at 4°C, a series of graded ethanol concentrations (varying from 30 to 100%) were used to dehydrate the cells. Samples were then air-dried, sputter-coated with gold, and examined by scanning electron microscope (Quanta FEG 250 FEI, Czech Republic), located at Egyptian Desert Research Center, Egypt.

### The effect of selected combination on bacterial membrane integrity

#### 260 nm absorbing material (nucleic acids)

The loss of nucleic acids through increased membrane permeability was assessed according to a previously published protocol^50^. Bacterial suspensions of *E. coli* and MRSA were prepared. Isolated colonies (5–7 colonies) of *E. coli* and MRSA in TSB were incubated at 37° C for 24 h. After incubation, the bacterial suspensions were centrifuged at 10.000 rpm for 5 min, washed twice with PBS then resuspended in saline to adjust the optical density at 600 nm to 1. Resuspended bacteria in saline were treated with ciprofloxacin (Ciprofloxacin 200 mg/100 mL vial, PHARCO) as a negative control, whereas cefotax (Cefotaxime 1000 mg/ 5 ml ampoule, EIPICO) was used as a positive control for *E. coli* and vancomycin (Vancomycin 500 mg/10 mL ampoule, Mylan) as a positive control for MRSA. The selected combination was tested at a final concentration equivalent to its MIC. Samples from bacterial cultures under each treatment were withdrawn after 30, 60 and 120 min, and were filtered through a 0.2-µm filter. After filtration, nucleic acid release was quantified at 260 nm using a UV–Visible Spectro nanophotometer (IMPLEN GmbH, Munich, Germany), Faculty of pharmacy, Cairo university, Cairo, Egypt^55^. The experiment was performed in triplicates and repeated on three independent times.

#### Potassium ions permeability

Bacterial suspensions of *E.coli* and MRSA were prepared according to a previously published protocol^50^. Isolated colonies (5–7 colonies) of *E. coli* and MRSA were inoculated in TSB, then incubated at 37° C for 24 h. Following incubation, bacterial cells were centrifuged at 10.000 rpm for 10 min and washed twice with PBS and resuspended in saline. The optical density was adjusted at 600 nm to 1. The bacterial cells were then treated with the MIC of the selected combination or kept untreated (negative control) for 6 h and centrifuged at 10.000 rpm for 10 min. After centrifugation, the amounts of potassium (K^+^) were measured in the supernatant using the ICP spectrometry technique^56^. Analyses were performed using a triple quadrupole ICP-MS system (iCAP TQ, Thermo Scientific) at the Inductively Coupled Plasma Unit (ICP) at the Central Laboratory for Elemental and Isotopic Analysis, Nuclear Research Center (NRC), Egyptian Atomic Energy Authority, Cairo, Egypt. The experiment was performed in triplicates and repeated on three independent times.

### The effect of the selected combination on the leakage of intracellular ions

The leakage of ions was assessed according to a previously published protocol^50^. Bacterial suspensions of *E. coli* and MRSA were prepared by inoculating 5–7 isolated colonies in TSB and incubating them at 37°C for 24 h. Following incubation, cells were centrifuged at 10.000 rpm for 10 min, washed twice with PBS, and resuspended in saline to an optical density of 1 at 600 nm. The bacterial cells were then treated with the MBC of the selected combination or kept untreated (negative control) for 6 h and then centrifuged. The concentration of phosphate (PO_4_^3–^) and Sulphur (S^2-^) ions in the supernatant was measured using the ICP spectrometry technique with triple quadrupole ICP-MS system (iCAP TQ, Thermo Scientific) at the Central Laboratory for Elemental and Isotopic Analysis, Nuclear Research Center, Egypt^56^. The experiment was performed in triplicates and repeated on three independent times.

### Preparation of different hydrogel formulations

A blank hydrogel was prepared by dissolving 1% carbopol (Sigma- Aldrich, Germany) in distilled water with continuous stirring, followed by tri-ethanolamine (Sigma- Aldrich, Germany) for stabilization and pH adjustment^57,58^. Similarly, the hydrogels for the tested agents were prepared at the MBC of each against tested MRSA strain, where combination of AgNPs-GTE loaded Cs-TPP-NPs was prepared at 4-MIC equivalent to MBC (1.25 / 312.5 µg/ml) and the MBC of ciprofloxacin against tested MRSA strain was at 62.5 µg/ml.

###  In vivo assessment of hydrogel efficacy in a murine diabetic skin infection lesion model

#### Experimental animals and design

Seventy male CD-1 mice (20–30 g of weight) were purchased from the laboratory animal facility of the Faculty of Pharmacy, Misr University for Science and Technology (MUST), 6th of October, Giza, Egypt. Mice were housed in a controlled environment to 22 ± 3 °C, 55 ± 5% humidity, and 12 h’ light/dark cycle. Animals were provided with a standard laboratory diet and water ad libitum. The mice were left to adapt to their environment for at least one week before starting the experiment. The total number of mice were divided in two separate experiments (n = 35). Mice were weighed and randomly distributed among five experimental groups (n = 7 mice / group / experiment).

The research procedures were carried out in compliance with the principles and recommendations of the Guidelines of the Care and Use of Laboratory Animals Association^59^ and ARRIVE guidelines^60^. All animal experiments were approved by the Research Ethics Committee (REC) of the Faculty of Pharmacy, Cairo University, Egypt #MI 2866 and Research Ethics Committee (REC) in National Center for Radiation Research and Technology (REC-NCRRT) with ethical clearance No: F/12C/25. Euthanasia was carried out in accordance with the American Veterinary Medical Association (AVMA) Guidelines for the Euthanasia of Animals^61^ and Guide for the Care and Use of Laboratory Animals^59^.

#### Induction of diabetes in mice

Experimental diabetes was induced by intraperitoneal injection of 150 mg/kg streptozotocin STZ (Molekula, Darlington, UK), dissolved in 0.05 M citrate buffer at a pH of 4.5^62^. A day before induction of diabetes, the mice were weighed, and individual doses were calculated. Basal blood glucose level was measured via a tail-vein blood sample using a One Touch Basic blood glucose monitoring system (ACCU-CHEK Instant Roche glucometer (Basel, Switzerland)) and recorded for each mouse before the start of chemical induction of diabetes. Immediately prior to injection, STZ was dissolved in sterile 50 mM sodium citrate buffer (pH 4.5) and injected intraperitoneally within 10 min. the mice were returned to their cages, provided with normal food and 10% sucrose water, and closely monitored every 2 h for 12 h for marked hypo activity, unresponsiveness, or convulsions. After 24 h, sucrose water was replaced with regular water. Five days’ post-injection, mice were fasted for 6 h, and blood glucose levels were measured again to ensure hyperglycemia. Mice with fasting blood glucose > 200 mg/dl were considered diabetic and included in subsequent experiments^62–64^.

#### Induction of skin infection lesion model in diabetic animals

The dorsal trunk area of the diabetic mice was shaved and depilated using an electrical hair clipper 24 h before induction of infection. Infection was induced by subcutaneously injecting with 100 μl of 4 × 10^9^ CFU of MRSA N315^65^. The mice then were returned to their cages and given food and water ad libitum. Following the induction of diabetes and subsequent subcutaneous infection with MRSA N315, mice were monitored for four days to allow for the development of characteristic staphylococcal skin lesions. The start of treatment was at day 4 post MRSA injection and day 9 after STZ injection (diabetes induction) and was defined as Day 0 (baseline). To account for the biological variability inherent in infection-induced lesions, treatment efficacy was quantified as the percentage of lesion contraction relative to the initial area measured for each individual mouse on Day 0.

The infection site was treated once daily for six days with 100 μl of either combination therapy (AgNPs-GTE loaded Cs-TPP-NPs) hydrogel at 4-MIC equivalent to MBC (1.25 / 312.5 μg/ml), unmedicated hydrogel as a vehicle control, or ciprofloxacin hydrogel (MBC = 62.5 μg/ml) as positive control^64,66,67^. These concentrations used for treatment were determined based on MIC, MBC, time kill assays and fractional inhibitory concentration index experiments results. The remain two groups where negative controls as one group was untreated, and the other group left healthy without induction of diabetes or infection. At the end of the experiment, the mice were euthanized by cervical dislocation performed by trained personnel, in accordance with the AVMA Guidelines for the Euthanasia of Animals (2020 Edition), under physical methods for small laboratory rodents (Section S2.2.2.3). Spleen and skin patches were then excised for viable bacterial counts, inflammatory markers assessment, and histopathological assay.

#### Evaluation of injury healing

The injury healing in terms of appearance and closure of lesions were assessed on daily basis from the photographs of the lesion area according to a previously published protocol^68^ . The lesion contraction (%) were calculated with the following equation:

$$Lesion \,\,contraction \left(\%\right)=\frac{\mathrm{l}\mathrm{e}\mathrm{s}\mathrm{i}\mathrm{o}\mathrm{n} \,\,\mathrm{a}\mathrm{r}\mathrm{e}\mathrm{a} \,\,\mathrm{o}\mathrm{n} \,\,\mathrm{d}\mathrm{a}\mathrm{y}\,\, \left(0\right)-\mathrm{l}\mathrm{e}\mathrm{s}\mathrm{i}\mathrm{o}\mathrm{n} \,\,\mathrm{a}\mathrm{r}\mathrm{e}\mathrm{a}\,\, \mathrm{o}\mathrm{n} \,\,\mathrm{d}\mathrm{a}\mathrm{y}\,\, (\mathrm{x})}{\mathrm{l}\mathrm{e}\mathrm{s}\mathrm{i}\mathrm{o}\mathrm{n}\,\, \mathrm{a}\mathrm{r}\mathrm{e}\mathrm{a}\,\, \mathrm{o}\mathrm{n} \,\,\mathrm{d}\mathrm{a}\mathrm{y} \,\,(0)}x 100$$

where $day (x)$ is any given time point and $day (0)$ is the initial time point or time zero.

#### Assessment of the  in vivo antimicrobial activity

Each excised skin tissue and spleen tissue was homogenized in saline by bead beating for 50 s 3 times, Part of saline homogenate was used for assessment of  in vivo antimicrobial activity of the tested combination (AgNPs-GTE loaded Cs-TPP-NPs) in comparison with other groups. Each homogenate was subjected to serial dilution. Subsequently, 10 μl of each dilution was spotted onto MSA plates. After 24 h of incubation at 37 °C, the colonies of MRSA N315 were counted to determine the viable count, expressed as CFU/ml^50^.

#### Assessment of anti-inflammatory activity

Nuclear factor Kappa B (NF-κB) levels in skin tissue homogenate was measured using the Mouse NF-κB ELISA Kit (MyBio Source, San Diego, CA, USA). Samples were prepared according to the kit manual and NF-κB concentration was determined and compared among different treatment groups^69^.

#### Histopathological assessment of skin specimen

Skin specimens were fixed in 10% formalin, trimmed off, washed, and dehydrated in ascending grades of alcohol. The dehydrated specimens were then cleared in xylene, embedded in paraffin blocks, and sectioned at 4–6 µm thick. The obtained tissue sections were deparaffinized using xylol and stained using hematoxylin and eosin (H&E) for histopathological examination through the electric light microscope^70^ with blinding of the operator. Histologic assessment and scoring was performed at Department of Pathology, Faculty of Veterinary Medicine, Cairo University according to a previous report^71^, where semi-quantitative method was used to evaluate the following histological processes and structures: re-epithelization, PMNL (polymorphonuclear leucocytes), fibroblasts, new vessels, and new collagen. Sections were evaluated according to the scale: 0, 1, 2, 3, 4 as mentioned in Table 1.

**Table 1:** Scale of semi-quantitative evaluation of histological sections.

Scale	0	1	2	3	4
Epithelization	Thickness of cut edges	Migration of cells (< 50%)	Migration of cells (≥ 50%)	Bridging the excision	Keratinization
Polymorphonuclear leucocyte	Absent	Mild surrounding tissue	Mild demarcation lines/granulation tissue	Moderate demarcation lines/granulation tissue	Marked demarcation lines/granulation tissue
Fibroblasts	Absent	Mild surrounding tissue	Mild- granulation tissue	Moderate- granulation tissue	Marked- granulation tissue
New vessels	Absent	Mild-subcutaneous tissue	Mild- granulation tissue	Moderate- granulation tissue	Marked- granulation tissue
Collagen	Absent	Minimal-granulation tissue	Mild- granulation tissue	Moderate- granulation tissue	Marked- granulation tissue

### Statistical analysis

Data were expressed as mean ± standard deviation (SD) unless otherwise specified. Statistical analyses were performed using GraphPad Prism 10.3.0 for Windows (GraphPad Software, San Diego, CA, USA). One-way analysis of variance (ANOVA) followed by Tukey’s multiple comparisons post-hoc test was used to determine statistical significance for  in vitro antibacterial activity, quantitative biofilm inhibition,  in vivo percentage of lesion contraction, bacterial load, and NFκB expression levels*.* For the time-kill kinetics assay and assessment of membrane integrity via nucleic acid leakage, a two-way ANOVA followed by Tukey’s multiple comparisons test was employed. An unpaired Student’s t-test was used to assess statistical differences in intracellular ion leakage and membrane integrity. Statistical significance was defined as follows: * *p* < 0.05, ** *p* < 0.01, *** *p* < 0.001, **** *p* < 0.0001 and ns (not statistically significant). All the experiments were performed in technical triplicates and repeated across three independent biological replicates.

## Results

### The γ radiation dose (7 kGy) completely decontaminated green tea

The microbial contamination of green tea (GT) was assessed before and after exposure to low doses of gamma radiation (1, 3, 5, and 7 kGy) by determining the total viable bacterial and fungal counts, expressed as colony-forming units per milliliter (CFU/ml. For bacterial growth, significant difference for bacterial count only detectable at 5 and 7 kGy (*P* value < 0.0001), as shown in (Supplementary Fig. 1A). Three pathogens: *Escherichia coli*, *Staphylococcus aureus*, and *Pseudomonas aeruginosa* were isolated from GT before exposure to γ irradiation, the viable count decreased gradually by increasing the radiation dose, becoming eliminated at 7 kGy. Meanwhile, *Salmonella* spp. and *Candida albicans* were not detected.

On the other hand, for fungal count, growth was detected in untreated GT samples then completely inhibited at doses (1, 3, 5 and 7 kGy). However, significant difference for fungal growth was noticed between 0.0 kGy and after exposure to any dose of gamma radiation (*P* < 0.0001), as shown in (Supplementary Fig. 1B). Therefore, 7 kGy, was chosen as the most effective dose for inhibiting all the microbial growth and leading to decontamination for GT sample.

### The γ radiation showed no alteration in the key components of green tea

As shown in (Supplementary Fig. 1C—1E), represent HPLC analysis for EGCG standard, GT before exposure to radiation, and after exposure to 7 kGy, respectively. The EGCG standard showed 2 prominent peaks at a retention time of 0.782 and 1.035 (Supplementary Fig. 1C). The freeze-dried GT extract before radiation showed 2 peaks at a retention time of 0.992 and 1.106 (Supplementary Fig. 1D). While, after exposure to 7 kGy (Supplementary Fig. 1E) the peaks remained at 0.991 and 1.106 confirming the presence of EGCG and indicating that gamma irradiation did not alter the key components of green tea.

### Synthesized AgNPs and ZnONPs showed polydispersed and spherical NPs in the nanoscale range

Formation of AgNPs was preliminary indicated by changing the color of the suspension to dark brown and detected by UV spectroscopy at 470nm (Supplementary Fig. 2A), while for ZnONPs, it was detected by UV spectroscopy at 335nm (Supplementary Fig. 2B). TEM image of synthesized NPs revealed the presence of polydispersed and spherical NPs of varying sizes in nanoscale ranging from 7 to 19nm for AgNPs and from 16 to 25nm for ZnONPs as displayed in (Supplementary Fig. 2C and D) respectively.

FTIR spectroscopy study was done for the synthesized NPs and the GTE separately. The relationship of the absorption bands (vibrational bands) with the chemical compounds in the sample was analyzed using the IR spectrum. This study was used to identify the biomolecules present in the GTE that are responsible for the reduction and stabilization processes of the ecofriendly synthesized nanoparticles as shown in (Supplementary Fig. 2E and F) for AgNPs and ZnONPs respectively. It was noted that, FTIR spectrum of GTE possesses absorption bands at 2923.60,1617.99, 1449.10, 1346.36, 1227.58, 1032.17, 822.79 and 611.19 cm^–1^. The band at 2923.60 cm^–1^ is due to the C–H stretch in alkanes. The strong band at 1617.99 cm^–1^ is attributed to the C = C stretch in aromatic ring and C = O stretch in polyphenols. The C–N stretch of amide-I in protein gives the band at 1449.10 cm^–1^. The C–O stretching in amino acid causes a band at 1032.17 cm^–1^. Finally, the weak band at 822.79 cm^–1^ is the result of the after effect of C–H out of plane bending. From the IR spectrum it can be noted that GTE sample is rich in polyphenols, carboxylic acid, polysaccharide, amino acid, and proteins. The presence of these biomolecules is involved in the reduction and stabilization (capping) actions of the synthesized NPs. Additionally, FTIR spectrum for the bio synthesized AgNPs has absorption bands at 3369.99, 2976.29, 1632.33, 1348.82, 1023.01, 578.20 and finally a new peak at 820.94 cm^-1^. Was found in the FTIR spectrum of AgNPs only, which may be due to conjugation of AgNPs with –OH groups as Ag–O. While for ZnONPs, the absorption bands can be appeared in the IR spectra as following, 2974.43, 1537.54, 1411.68, 1036.58, and 820.04 cm^-1^. In addition to the absorption bands of these biomolecules, two new peaks appearing at 690.06 and 514.39 cm^–1^ in the IR spectrum of the ZnONPs are characteristic for Zn–O bond which confirms the formation of ZnO nanoparticles.

Average particle size was determined by dynamic light scattering (DLS), where the mean is defined as 46.3 nm and 67.6 nm for AgNPs and ZnONPs respectively, (Supplementary Fig. 3A and B). The average zeta potential value for biologically synthesized AgNPs and ZnONPs was -4.54mV and -4.52mV respectively, (Supplementary Fig. 3C and D). The XRD pattern to the AgNPs was presented in (Supplementary Fig. 3E); many peaks were observed, those for AgNPs. Diffraction characteristics are showing within 2θ (degree) as 38.13º, 44.21º, 64.47º, and 77.37º where these peaks describe the Bragg’s reflections (111), (200), (220) and (311) planes in that order respectively, and these data revealed crystalline nature of AgNPs. Similarly, The XRD pattern of the ZnONPs exhibited characteristic diffraction peaks at 2θ values corresponding to the (100), (002), (101), (102), (110), (103), and (112) planes, confirming the formation of a hexagonal wurtzite structure as shown in (Supplementary Fig. 3F).

The elemental mapping of AgNPs and ZnONPs was shown in (Supplementary Fig. 4A and C). The models are designated as Ag, C, and O and Zn, C, and O respectively, based on their distribution. In addition, the GTE was represented by the atoms C and O. As shown in the figure, the AgNPs and ZnONPs were then uniformly dispersed over the GTE atoms (C, and O). The quantitative elemental structure of greenly synthesized AgNPs and ZnONPs was assessed by EDS analysis, as shown in (Supplementary Fig. 4B and D). Both EDS spectrums confirmed the successful formation of AgNPs and ZnONPs, showing a characteristic peak of silver and zinc at around 3 keV and 1 keV respectively. Additional signals corresponding to carbon and oxygen were also detected, which may be attributed to the organic phytochemicals from the GTE acting as reducing and stabilizing agent. Moreover, the presence of O in the spectrum of ZnONPs indicated the formation of zinc oxide.

### Synthesized GTE loaded Cs-TPP NPs showed spherical NPs in the nanoscale range

TEM analysis of synthesized green tea extract loaded chitosan tripoly phosphate nanoparticles (GTE loaded Cs-TPP-NPs) revealed the presence of homogenously spherical nanoparticles of varying sizes in nanoscale ranging from 10 to 23 nm as displayed in (Supplementary Fig. 5A). Average particle size was determined by DLS method and representative size distribution graph is shown in (Supplementary Fig. 5B), where the mean is defined as 39 nm. The FTIR spectra were used to characterize the functional groups and to determine the molecular interaction between the synthesized GTE loaded Cs-TPP-NPs and GTE. It was noted that, FTIR spectrum of GTE possesses absorption bands at 3649.63, 2981.12, 2884.06, 1629, 1449.12, 1364.54, 1215.44 and 1035.63 cm^-1^. Additionally, FTIR spectrum for the bio synthesized GTE loaded Cs-TPP-NPs has absorption bands at 3365.90, 2977.59, 2886.33, 1563.44, 1384.54 and 1063.04 cm^-1^ as shown in (Supplementary Fig. 5C). The broad peak at 3365.90 cm^-1^ assigned to O–H stretching of hydroxyl group and the peak at 2977.59 cm^-1^ corresponds to C-H bonds stretching of alkanes. The peak at 2886.33 assigned to O–H of carboxylic acid. A new peak appeared at 1563.44 corresponds to phosphoric groups of TPP interacting with the ammonium groups of chitosan. The bands at 1629 cm^-1^ and 1449.12 appeared in the spectrum of GTE only assigned to flavonoid C = O functional groups and C = C of aromatic ring respectively. The peak at 1384.54 is characteristic for hydroxyl group and phenolic hydroxyl. Peak found at 1215.44 cm^-1^ in the spectrum of GTE only refers to C-N of aliphatic amines. Finally, the peak at 1063.04 cm^-1^ corresponds to C-O stretching vibrations in amino acids. The XRD spectrum of GTE loaded Cs-TPP-NPs in (Supplementary Fig. 5D) exhibited non-crystalline behavior (amorphous nature), in which a broad peak was present at 2θ = 22°, no intense peaks of the GTE nor TPP were visible, indicating that there has been a successful cross-linking between chitosan and TPP along with the dispersion of GTE within the nanoparticles. Finally, the average zeta potential value for GTE loaded Cs-TPP-NPs was 21.6 mV (Supplementary Fig. 5F).

### AgNPs showed best antibacterial activity against tested bacteria among the screened preparations

**Fig. 1:**
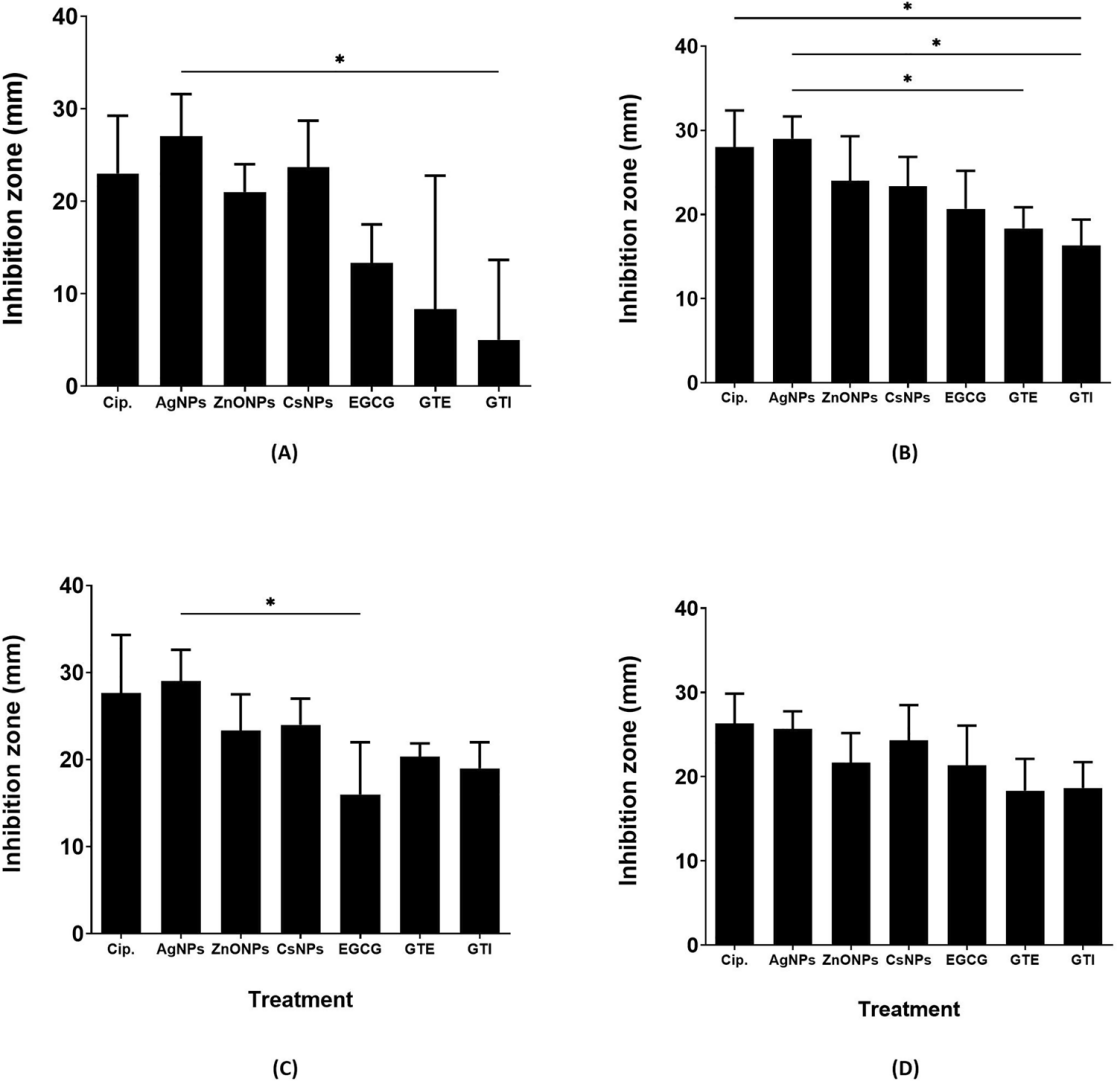
In vitro antibacterial activity of the synthesized nanoparticle (NP) formulations. Inhibition zones (mm) of AgNPs, ZnONPs, and GTE loaded Cs-TPP-NPs, alongside with EGCG, GTE and GTI against (**A**) *Pseudomonas aeruginosa*, (**B**) *E. coli*, (**C**) MRSA and (**D**) *Streptococcus pyogenes.* Assay was performed using the agar well diffusion method with Ciprofloxacin as the positive control. Data are represented as means of inhibition zones (mm) ± SD (n = 3 independent biological experiments, each performed with three technical replicates). Statistical significance was determined by one-way ANOVA followed by Tukey’s multiple comparisons test (*P < 0.05). The statistical analysis was performed using GraphPad Prism (version 10.3.0).

Evaluation of antimicrobial activity of different preparations by using the agar well-diffusion method against *P. aeruginosa*, *E. coli*, MRSA, and *S. pyogenes* revealed that, AgNPs and GTE loaded Cs-TPP-NPs exhibited the largest inhibition zones among the screened preparations against *P. aeruginosa* with significant difference between AgNPs and GTI (*p* < 0.05) (Fig. 1A). Similarly, AgNPs showed the highest inhibition against *E. coli*, significantly outperforming GTE and GTI (*p* < 0.05) (Fig. 1B). Against MRSA, AgNPs continued to demonstrate the strongest antibacterial activity, maintaining its superior efficacy as observed against other pathogens. It exhibited the largest inhibition zones, with a significant difference compared to EGCG (*p* < 0.05) (Fig. 1C). All preparations exhibited a comparable inhibitory effect against *S. pyogenes*, with no significant differences observed among them (Fig. 1D).

### AgNPs had the lowest MIC and MBC values among the screened preparations

Silver nanoparticles (AgNPs) had the lowest concentration among all preparations in inhibiting *P. aeruginosa*, *E. coli*, MRSA and *S. pyogenes* growth with MIC values of 0.039, 1.25, 0.625 and 0.625 µg/ml, respectively followed by ZnONPs with MIC values of 3.5, 1.75, 1.75 and 1.75 µg/ml against same bacterial strains. Both exhibited higher antibacterial activity than ciprofloxacin (as positive control), EGCG, GTE, and GTI (as negative controls).

As shown in Table 2, AgNPs also exhibited the highest bactericidal activity among the tested preparations against *P. aeruginosa*, *E. coli*, MRSA and *S. pyogenes* with MBC values ranged between 0.156 µg/ml and 1.25 µg/ml. While the next higher potent bactericidal preparation was ZnONPs with MBC values between 3.5 µg/ml to 7 µg/ml. In contrast, the positive control, ciprofloxacin, showed significantly weaker bactericidal activity, with MBC values of 62.5 µg/mL for *P. aeruginosa*, MRSA, and *S. pyogenes* and 31.25 µg/mL for *E. coli*. GTE-loaded Cs-TPP-NPs demonstrated moderate bactericidal effects with all tested standard strains, with MBC values ranging from 625 to 1250 µg/mL, though only bacteriostatic activity against *E. coli*. GTE and GTI displayed the weakest antimicrobial activity, remaining bacteriostatic against most pathogens (Table 2).

**Table 2:** Determination of MIC and MBC of different preparations.

**Bacteria**	*Pseudomonas aeruginosa*			*E. coli*			MRSA			*Streptococcus pyogenes*		
	MIC µg/ml	MBC µg/ml	Ratio MBC/MIC	MIC µg/ml	MBC µg/ml	Ratio MBC/MIC	MIC µg/ml	MBC µg/ml	Ratio MBC/MIC	MIC µg/ml	MBC µg/ml	Ratio MBC/MIC
**Treatment**												
Ciprofloxacin	15.6	62.5	4	15.6	31.25	2	31.25	62.5	2	15.6	62.5	4
AgNPs	0.039	0.156	4	1.25	1.25	1	0.625	1.25	2	0.625	1.25	2
ZnONPs	3.5	7	2	1.75	3.5	2	1.75	3.5	2	1.75	7	4
GTE loaded Cs-TPP-NPs	312.5	1250	4	156.25	1250	8	312.5	1250	4	312.5	625	2
EGCG	1250	ND	ND	625	2500	4	625	ND	ND	2500	2500	1
GTE	40,000	ND	ND	5000	ND	ND	2500	ND	ND	5000	20,000	4
GTI	All turbid	ND	ND	12,500	ND	ND	25,000	50,000	2	50,000	50,000	1

MIC: minimum inhibitory concentration, MBC: minimum bactericidal concentration,

Ratio MBC/MIC interpretation ≤ 4 bactericidal and ˃ 4 bacteriostatic.

ND: Not detected.

The experiment was performed in triplicates and repeated on three independent times.

### AgNPs showed significant biofilm inhibition among the screened preparations

**Fig. 2:**
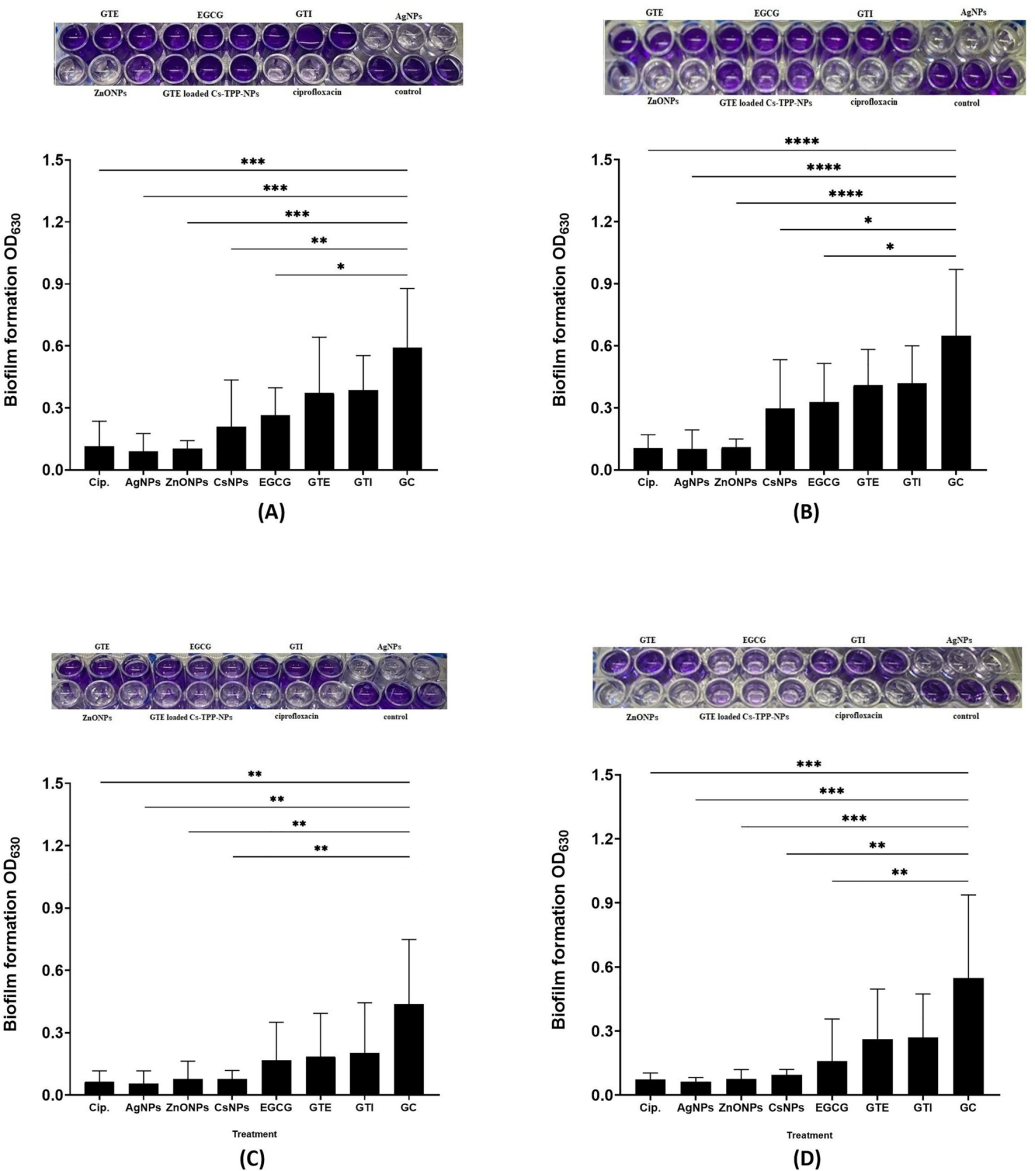
Quantitative and visual assessment of biofilm inhibition. Absorbance-based quantification of biofilm biomass following exposure to sub-MIC concentrations of nanoparticle formulations (AgNPs, ZnONPs, and GTE loaded Cs-TPP-NPs) and plant-derived compounds (EGCG, GTE, and GTI) against (**A**) *P. aeruginosa*, (**B**) *E. coli*, (**C**) MRSA and (**D**) *S. pyogenes*. Representative photographs illustrate crystal violet-stained biofilms in 96-well microtiter plates. Ciprofloxacin served as the positive control. Data are expressed as mean ± SD, (n = 3 independent biological experiments, each performed with three technical replicates). Statistical significance was determined by one-way ANOVA followed by Tukey’s multiple comparison test (* *P* < 0.05, ** *P* < 0.01, *** *P* < 0.001, **** *P* < 0.0001). Statistical analysis was performed using GraphPad Prism software (version 10.3.0).

Biofilm production is considered as a marker of virulence. The effect of AgNPs, ZnONPs, GTE loaded Cs-TPP-NPs, EGCG, GTE and GTI on the *in-vitro* biofilm formation of the selected standard bacterial strains (*P. aeruginosa*, *E. coli*, MRSA and *S. pyogenes*) in comparison with ciprofloxacin and growth control were studied using sub-MIC concentrations (1/2 MIC). Results showed that all the tested bacterial strains were biofilm producers. AgNPs, ZnONPs, GTE-loaded Cs-TPP-NPs, ciprofloxacin, and EGCG demonstrated significant antibiofilm activity against *P. aeruginosa*, *E. coli*, MRSA, and *S. pyogenes*, as indicated by the reduction in the average OD_630_ measuring crystal violet dye bound to biofilm mass. In contrast, GTE and GTI showed no significant effect. The level of significance for each preparation is detailed in (Fig. 2A–D).

### AgNPs showed the lowest time-to-kill among the screened preparations

**Fig. 3:**
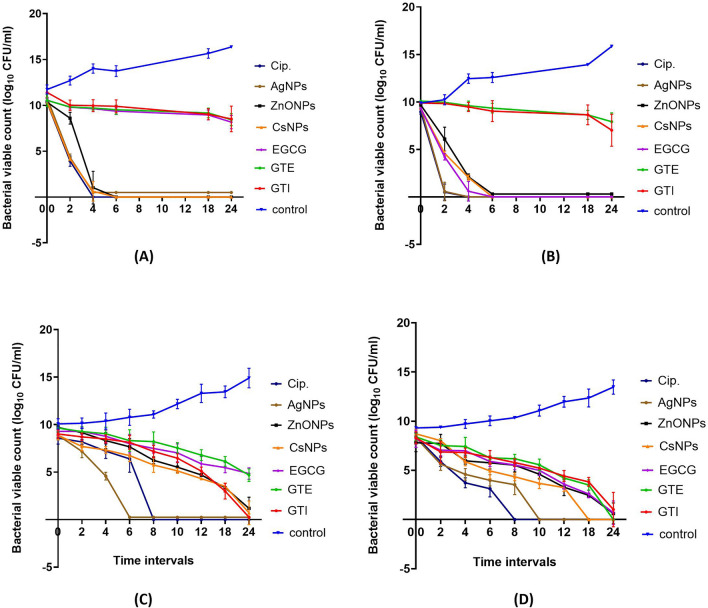
Time- kill kinetics of nanoparticle formulations. Bacterial survival curves for (**A**) *P. aeruginosa*, (**B**) *E. coli*, (**C**) MRSA, and (**D**) *S. pyogenes* following exposure to the MBC of AgNPs, ZnONPs, GTE loaded Cs-TPP-NPs, EGCG, GTE and GTI. Ciprofloxacin and untreated cells served as positive and negative controls, respectively. Data are presented as mean CFU/ml ± SD at each time point (n = 3 independent biological experiments, each performed with three technical replicates). Statistical significance was determined by ordinary two-way ANOVA. Statistical analysis was performed using GraphPad Prism software (version 10.3.0).

The killing kinetics of various preparations against *P. aeruginosa*, *E. coli*, MRSA and *S. pyogenes* were evaluated at their respective MBC concentrations (Fig. 3 A-D). *P. aeruginosa* was eradicated within 4 h by ciprofloxacin (62.5 µg/ml), AgNPs (0.156 µg/ml), ZnONPs (7 µg/ml), and GTE-loaded Cs-TPP-NPs (1250 µg/ml), while EGCG, GTE, and GTI exhibited no bactericidal activity up to 24 h. *E. coli* was killed within 2 h by ciprofloxacin (31.25 µg/ml) and AgNPs (1.25 µg/ml), whereas ZnONPs (3.5 µg/ml), GTE-loaded Cs-TPP-NPs (1250 µg/ml), and EGCG (2500 µg/ml) required 4 h, with GTE and GTI showing no killing effect until 24 h. MRSA was killed by AgNPs (1.25 µg/ml) after 6 h, ciprofloxacin (62.5 µg/ml) within 8 h, and ZnONPs (3.5 µg/ml) and GTI (50,000 µg/ml) after 24 h while EGCG and GTE exhibited no bactericidal effect. *S. pyogenes* was eliminated after 10 h by ciprofloxacin 62.5 µg/ml) and AgNPs (1.25 µg/ml), while GTE-loaded Cs-TPP-NPs (625 µg/ml) required 12 h; ZnONPs (7 µg/ml), EGCG (2500 µg/ml), GTE (20,000 µg/ml), and GTI (50,000 µg/ml) needed 24 h for complete bacterial eradication.

### The GTE loaded Cs-TPP NPs/AgNPs showed a synergistic effect against tested bacteria

Different combinations between preparations including GTE with AgNPs, ZnONPs and GTE loaded Cs-TPP-NPs as well as GTE loaded Cs-TPP NPs with AgNPs and ZnONPs were tested in a 1:1 ratio against the four standard bacterial strains: *P aeruginosa*., *E. coli*, MRSA and *S pyogenes*. to evaluate their antimicrobial efficacy. The GTE/AgNPs combination exhibited a synergistic effect against *P aeruginosa*., *E. coli*, MRSA and *S pyogenes*. with ƩFIC value of 0.488, 0.047, 0.094 and 0.078 respectively. GTE/ZnONPs showed an indifference effect against *P aeruginosa*. (ƩFIC = 1.125), an additive effect against *E. coli* and MRSA (ƩFIC value of 0.75 and 1 respectively) and a synergistic effect against *S pyogenes*. (ƩFIC value = 0.375). The GTE/GTE loaded Cs-TPP-NPs combination revealed an indifference effect against *P aeruginosa*., *E. coli* and *S pyogenes*. (ƩFIC = 2.125, 2.5, and 1.5, respectively), while displaying an additive effect against MRSA (ƩFIC = 1). Meanwhile, GTE loaded Cs-TPP NPs/AgNPs demonstrated a synergistic effect against all four pathogens with ƩFIC values of 0.5 against *P aeruginosa*., and 0.187 against *E. coli*, MRSA and *Ss pyogenes*.. Finally, the GTE loaded Cs-TPP NPs/ZnONPs combination exhibited an indifference effect against *P aeruginosa*., *E. coli* and *S pyogenes*. with (ƩFIC = 1.5, 1.5 and 2, respectively) and an additive effect against *MRSA* (ƩFIC = 1) (Table 3).

**Table 3:** Determination of synergism/ antagonism and fractional inhibitory concentration index (ƩFIC) of different combinations.

Treatments		MIC_a_ µg/ml	MIC_b_ µg/ml	MIC_a+_ µg/ml	MIC_b+_ µg/ml	ƩFIC	Interpretation
a	b						
*P. aeruginosa*							
GTE	AgNPs	40,000	0.039	39	0.019	0.488	S
	ZnONPs	40,000	3.5	5000	3.5	1.125	I
	GTE loaded Cs-TPP-NPs	40,000	312.5	5000	625	2.125	I
GTE loaded Cs-TPP NPs	AgNPs	312.5	0.039	4.88	0.019	0.503	S
	ZnONPs	312.5	3.5	312.5	1.75	1.5	I
*E. coli*							
GTE	AgNPs	5000	1.25	78	0.039	0.047	S
	ZnONPs	5000	1.75	1250	0.875	0.750	A
	GTE loaded Cs-TPP-NPs	5000	156.3	2500	312.5	2.5	I
GTE loaded Cs-TPP NPs	AgNPs	156.3	1.25	19.5	0.078	0.187	S
	ZnONPs	156.3	1.75	156.3	0.875	1.5	I
**MRSA**							
GTE	AgNPs	2500	0.625	78	0.039	0.094	S
	ZnONPs	2500	1.75	1250	0.875	1	A
	GTE loaded Cs-TPP-NPs	2500	312.5	1250	156.3	1	A
GTE loaded Cs-TPP NPs	AgNPs	312.5	0.625	19.5	0.078	0.187	S
	ZnONPs	312.5	1.75	156.3	0.875	1.0	A
**S. pyogenes**							
GTE	AgNPs	5000	0.625	78	0.039	0.078	S
	ZnONPs	5000	1.75	625	0.438	0.375	S
	GTE loaded Cs-TPP-NPs	5000	312.5	2500	312.5	1.5	I
GTE loaded Cs-TPP NPs	AgNPs	312.5	0.625	19.5	0.078	0.187	S
	ZnONPs	312.5	1.75	312.5	1.75	2.0	I

The experiment was performed in triplicates and repeated on three independent times.

MICa and MICb: MIC of each individual component a and b, respectively; MICa + and MICb + : MIC of each component a and b in the combination, respectively. The ΣFIC for each combination was interpreted as ≤ 0.5 representing synergy (S); > 0.5–1.0 as additive; > 1.0 ≤ 4.0 representing indifference (I); and > 4.0 representing antagonism (A).

### AgNPs-GTE loaded Cs-TPP-NPs combination distorted cell wall and membrane of *E. coli* and MRSA

**Fig. 4:**
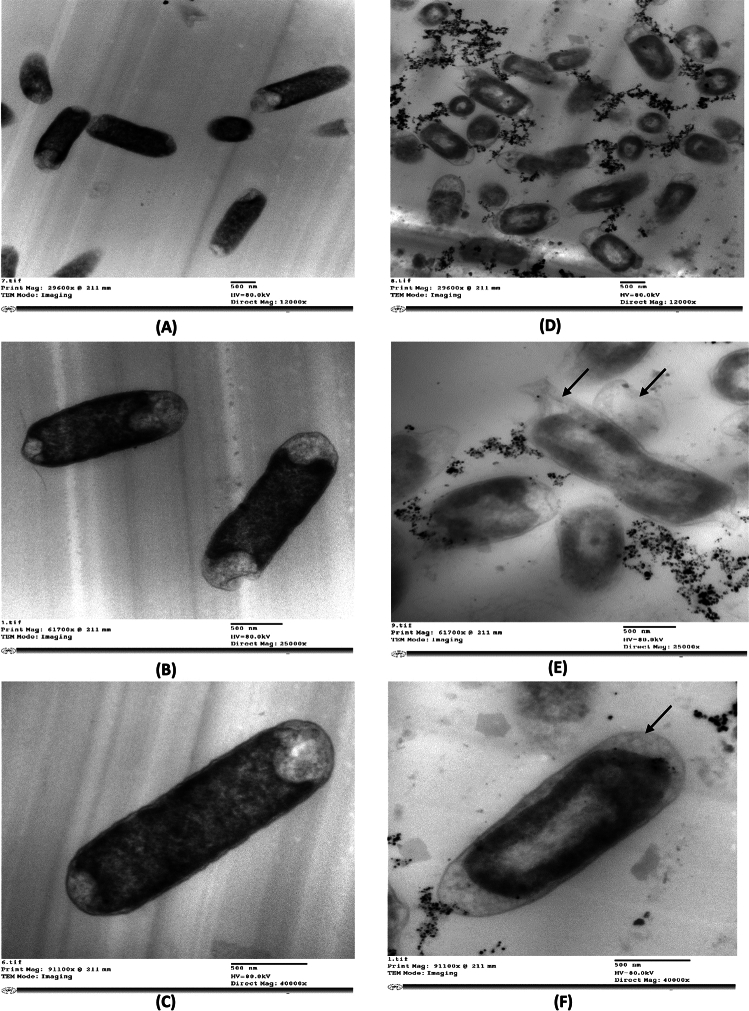
Transmission electron microscopy (TEM) micrographs of morphological changes in *E. coli.* (**A**–**C**) Untreated control *E. coli* cells displaying characteristic rod-shaped morphology, intact cell walls, and dense cytoplasm. (**A**: 12,000x; **B**: 25,000x; **C**: 40,000x). (**D**–**F**) *E. coli* treated with the AgNPs-GTE-loaded Cs-TPP-NPs combination, showing severe morphological damage. Observed cell changes include cell wall and membrane distortion, cytoplasmic shrinkage, separation of the plasma membrane from the cell wall, and leakage of intracellular contents (**D**: 12,000x; **E**: 25,000x; **F**: 40,000x). Micrographs are representative of multiple fields of view. Imaging was performed using a JEOL JEM-1400 Flash electron microscope at 120 kV, with a resolution of 0.2 nm (JEOL Ltd., Tokyo, Japan).

**Fig. 5:**
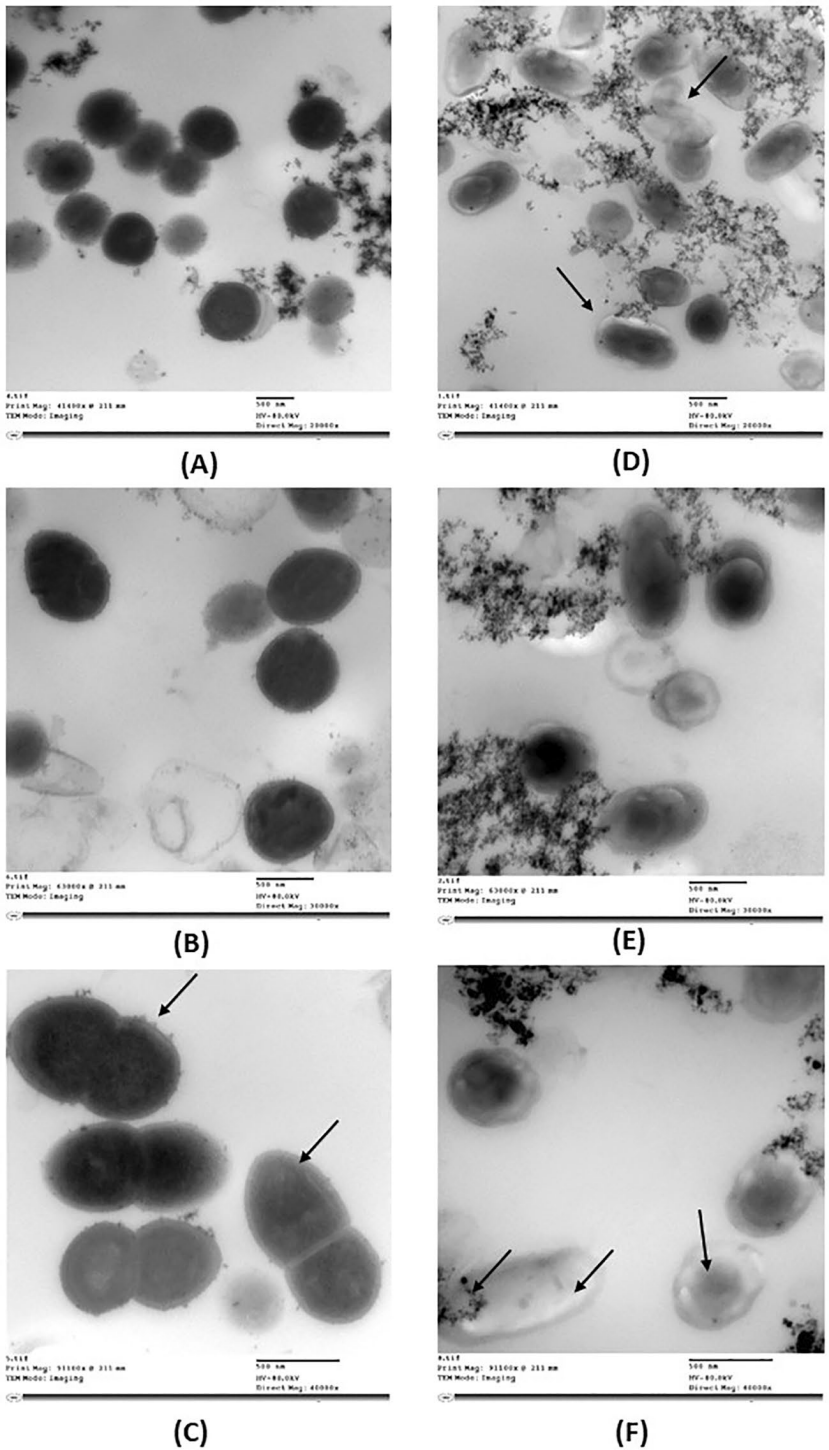
Transmission electron microscopy (TEM) micrographs of morphological changes in methicillin-resistant *Staphylococcus aureus* (MRSA). (**A**–**C**) Untreated control MRSA cells revealed characteristic spherical morphology, thick cell walls, and well-preserved intracellular structures including detailed view of dividing and non-dividing cells (**A**: 12,000x; **B**: 25,000x; **C**: 40,000x). (**D**–**F**) MRSA treated with the AgNPs-GTE-loaded Cs-TPP-NPs combination, showing severe morphological damage. Observed alterations include loss of spherical shape, cell lysis, reduced cytoplasmic density (“hollow cell” formation), disruption of the peptidoglycan layer, separation of the plasma membrane from the cell wall, visible deposition of AgNPs inside the bacterial cells, and leakage of cytoplasmic contents (**D**: 12,000x; **E**: 25,000x; **F**: 40,000x). Micrographs are representative of multiple fields of view. Imaging was performed using a JEOL JEM-1400 Flash electron microscope at 120 kV, with a resolution of 0.2 nm (JEOL Ltd., Tokyo, Japan).

Exhibiting superior activity, the GTE-loaded Cs-TPP-NPs and AgNPs combination was examined for its potential mechanisms of action through multiple assays, including induced morphological alterations in the tested bacteria, loss of integrity and leakage of intracellular components. The mechanism of action of AgNPs-GTE loaded Cs-TPP-NPs combination on membrane permeability was shown in TEM photographs of *E. coli* and MRSA (Figs. 4 and 5). Before treatment, both cells exhibited well-defined, intact structures with preserved intracellular contents and normal morphology (Fig. 4A-C) and (Fig. 5A-C). Exposure to AgNPs-GTE loaded Cs-TPP-NPs combination at its MBC induced significant morphological alterations including shrinkage of bacterial cells with distortion of cell wall and cell membrane. Some cells displayed hollow, disfigured cell walls due to cytoplasmic release, while others exhibited membrane detachment from the cell wall (Fig. 4D-F) and (Fig. 5D-F).

### AgNPs-GTE loaded Cs-TPP-NPs combination inhibited the biofilm formation

**Fig. 6:**
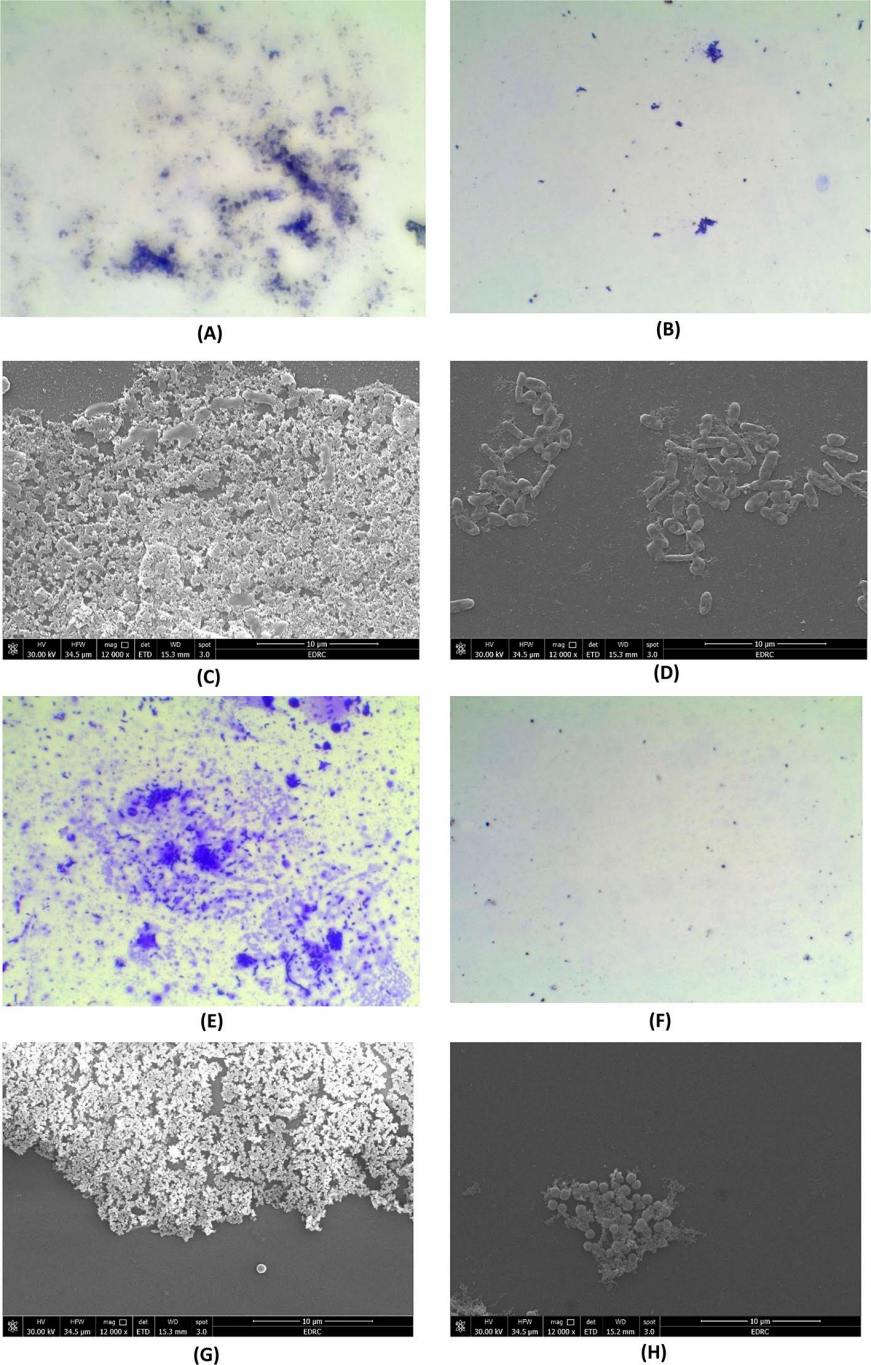
Microscopic visualization of antibiofilm activity. Light microscopy and scanning electron microscopy (SEM) images of *E. coli* and MRSA biofilms. (**A**, **C**) Untreated control *E. coli* biofilms under light microscopy and SEM, respectively; (**B**, **D**) *E. coli* biofilms treated with the AgNPs-GTE-loaded Cs-TPP-NPs combination at 1/2 MIC. (**E**, **G**) Untreated control MRSA biofilms under light microscopy and SEM, respectively; (**F**, **H**) MRSA biofilms treated with the combination at 1/2 MIC. Panels (**A**, **B**, **E**, **F**) represent light microscopy, while (**C**, **D**, **G**, **H**) represent SEM. Untreated controls exhibit robust biofilm architecture and high cellular adhesion, whereas nanoparticle treated samples demonstrate a marked reduction in biomass and cellular density showing biofilm inhibition. Crystal violet-stained biofilms were examined under a bright-field microscope (Euromex iScope series, 110–240 V / 50–60 Hz) at 40 × magnification and imaged using an attached digital camera. SEM imaging was performed using a Quanta FEG 250 (FEI, Czech Republic) at 12,000 × magnification.

Microscopic technique was used to qualitatively analyze biofilm architecture of *E. coli* and MRSA strains. In light microscopic analysis, an aggregated biofilm formation was observed in control samples (Fig. 6A and E) for *E. coli* and MRSA respectively, while in treated samples, a significant reduction in the biofilm covered surface area was observed (Fig. 6B and F) for *E. coli* and MRSA respectively.

The SEM was chosen for analyzing the surface and morphological changes in biofilm cells exposed to the selected combination. Regarding *E. coli* and MRSA biofilm formation, cellular adhesion and aggregation were detected in the untreated biofilm (control) (Fig. 6C and G) for *E. coli* and MRSA respectively; however, for biofilm treated with AgNPs-GTE loaded Cs-TPP-NPs, a lower number of adherent cells were observed (Fig. 6D and H) for *E. coli* and MRSA respectively.

### AgNPs-GTE loaded Cs-TPP-NPs combination affected membrane integrity of tested bacteria

#### Increased the leakage of 260 nm absorbing material (nucleic acids)

**Fig. 7:**
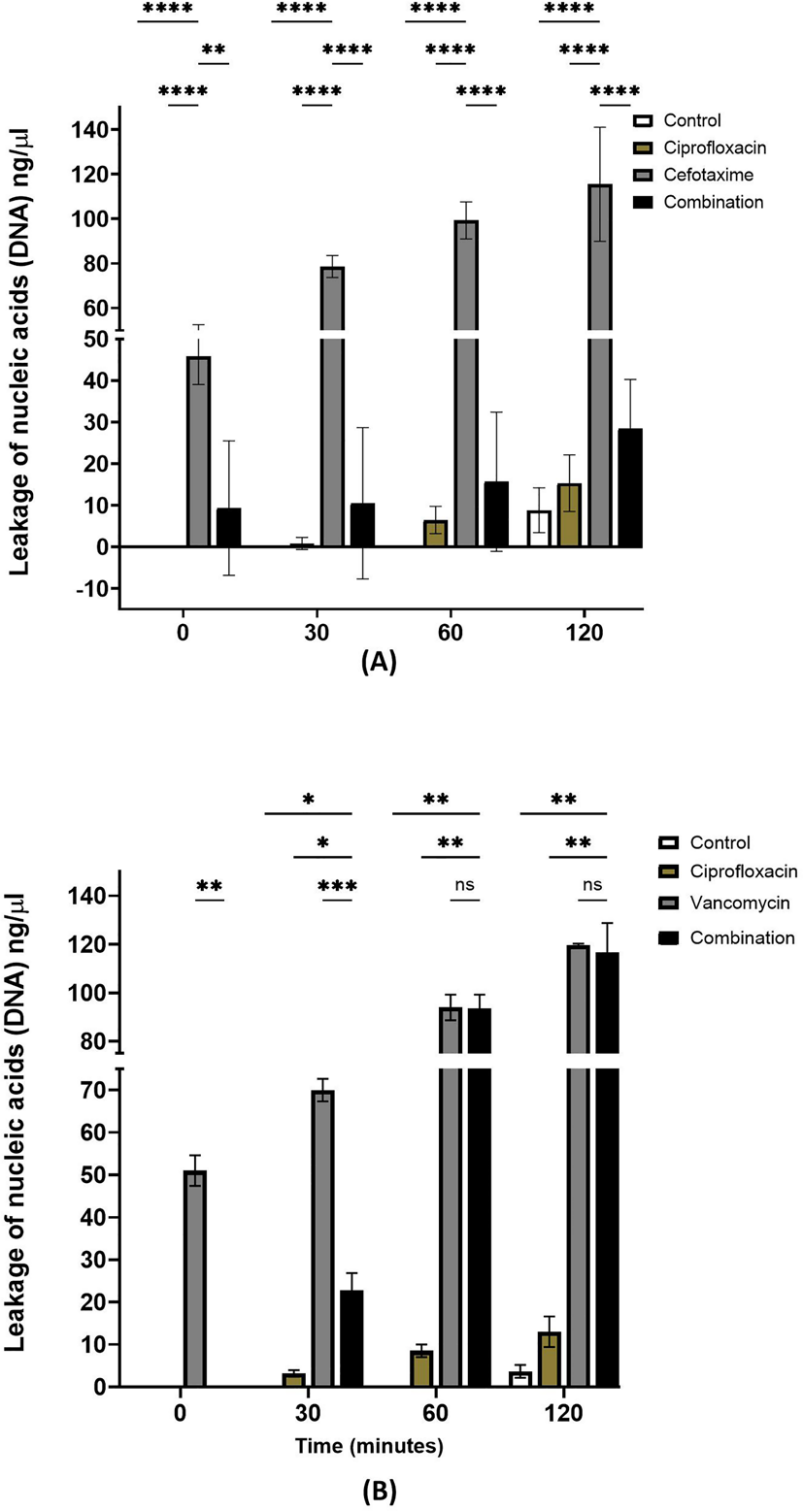
Assessment of membrane integrity via nucleic acid leakage. Concentration of extracellular nucleic acids in filtrates of (**A**) *E. coli* and (**B**) MRSA following treatment with the AgNPs-GTE-loaded Cs-TPP-NPs combination (black bars) compared to ciprofloxacin, cefotaxime, vancomycin, and untreated controls confirmed membrane damage induced by the NPs combination. Data are expressed as mean ± SD (n = 3 independent biological experiments, each performed with three technical replicates). Statistical significance was determined by two-way ANOVA followed by Tukey’s multiple comparisons test (* *P* < 0.05, ** *P* < 0.01, *** *P* < 0.001, **** *P* < 0.0001; ns: not significant). Statistical analysis was performed using GraphPad Prism software (version 10.3.0).

The amount of released nucleic acids of filtrates from control (untreated bacterial suspension), negative control (ciprofloxacin) and treated AgNPs-GTE loaded Cs-TPP-NPs combination suspensions were not significantly different after 30, 60 and 120 min against *E. coli* (Fig. 7A). For MRSA, nucleic acid leakage progressively increased over time, becoming significantly higher than the negative control (ciprofloxacin) after 30 min with *P value* < 0.0001. By 90 and 120 min, it reached levels comparable to the positive control (vancomycin), with no significant difference observed (Fig. 7B).

#### Increased potassium ions (K +) permeability

**Fig. 8:**
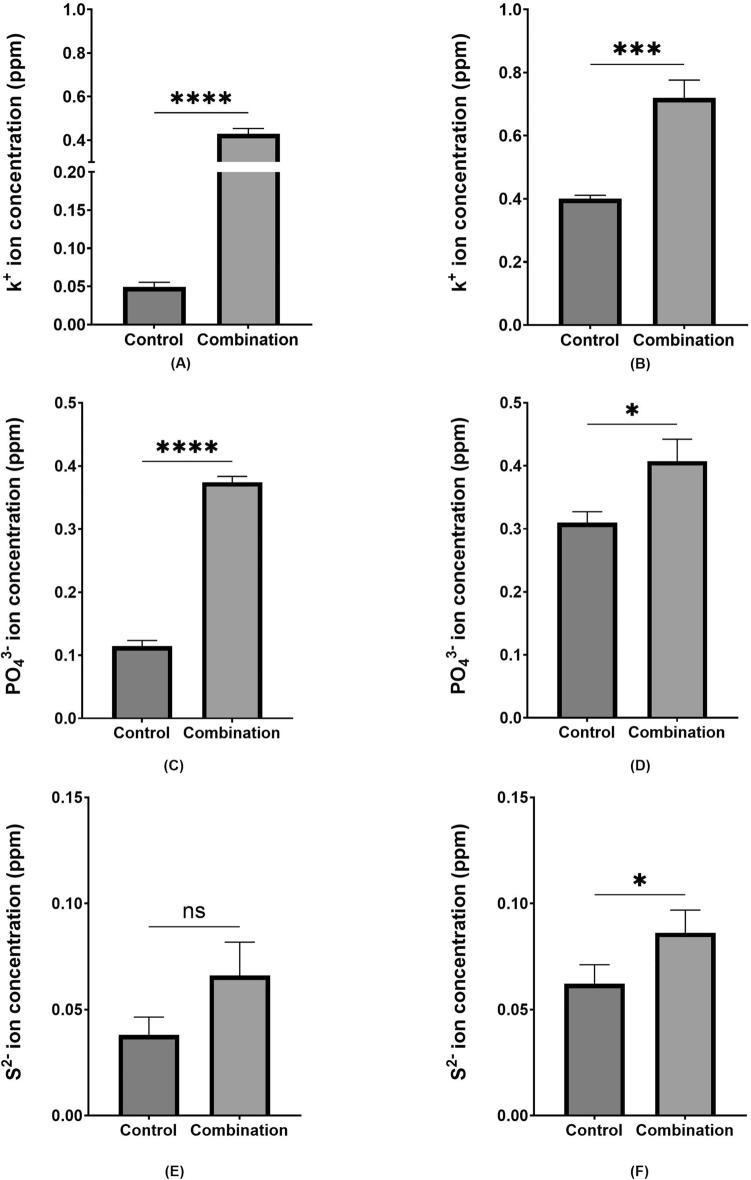
Effect of AgNPs-GTE-loaded Cs-TPP-NPs combination on intracellular ion leakage and membrane integrity. (**A**, **B**) Potassium ion (K⁺) levels in (**A**) *E. coli* and (**B**) MRSA cells following treatment with the AgNPs-GTE-loaded Cs-TPP-NPs combination resulted in a significant increase in extracellular K⁺ levels compared with the untreated control, indicating disruption of bacterial membrane integrity. (**C**–**F**) Extracellular concentrations of phosphate (PO₄^3^⁻) and sulfur (S^2-^) ions in (**C**, **E**) *E. coli* and (**D**, **F**) MRSA, before and after 6 h of treatment compared with the untreated control. Data are expressed as mean ± SD (n = 3 independent biological experiments, each performed with three technical replicates). Statistical significance was determined by an unpaired Student’s t-test (* *p* < 0.05, *** *p* < 0.001, **** *p* < 0.0001; ns means non-significant). Statistical analysis was performed using GraphPad Prism software (version 10.3.0).

The effect of AgNPs-GTE loaded Cs-TPP-NPs combination on the membrane integrity of bacterial cells was assessed through the leakage of potassium ions (k^+^) Fig. 8A and B. Treatment of *E. coli* and MRSA with AgNPs-GTE loaded Cs-TPP-NPs combination at its MBC for 6 h has led to a significant observed leakage of k^+^ compared with the untreated control with *P value* < 0.0001.

### AgNPs-GTE loaded Cs-TPP-NPs combination increased the leakage of intracellular ions

The effect of adding AgNPs-GTE loaded Cs-TPP-NPs combination at its MBC on the phosphate (PO_4_^3-^) and sulfur (S^2-^) leakage in both *E. coli* and MRSA is shown in (Fig. 8C-F). Compared to the control (untreated bacterial suspensions), leakage was observed after AgNPs-GTE loaded Cs-TPP-NPs combination addition. These results suggested that increased membrane permeability is a factor in the mechanism of antimicrobial action.

### AgNPs-GTE loaded Cs-TPP-NPs hydrogel healed diabetic skin infection lesions

The most promising formulation AgNPs-GTE loaded Cs-TPP-NPs combination was tested in the diabetic murine skin of MRSA infection lesion model due to their potent antimicrobial activity in vitro. A single intraperitoneal injection of STZ induced type 1 diabetes in mice, with blood glucose levels rising to ≥ 200 mg/dl within 5–7 days, accompanied by symptoms of polydipsia and polyuria. Ninety-six hours’ post-subcutaneous infection with MRSA N315, skin lesions were observed. The diabetic murine skin infection lesion model was accepted since skin lesions represent a hallmark of staphylococcal infections. Based on the results of MIC, MBC, time kill assay and fractional inhibitory concentration index experiments, the infection sites were then treated for six days with either AgNPs-GTE loaded Cs-TPP-NPs combination hydrogel at 4-MIC equivalent to MBC, ciprofloxacin hydrogel as positive control, blank vehicle hydrogel as negative control or left untreated as negative control.

**Fig. 9:**
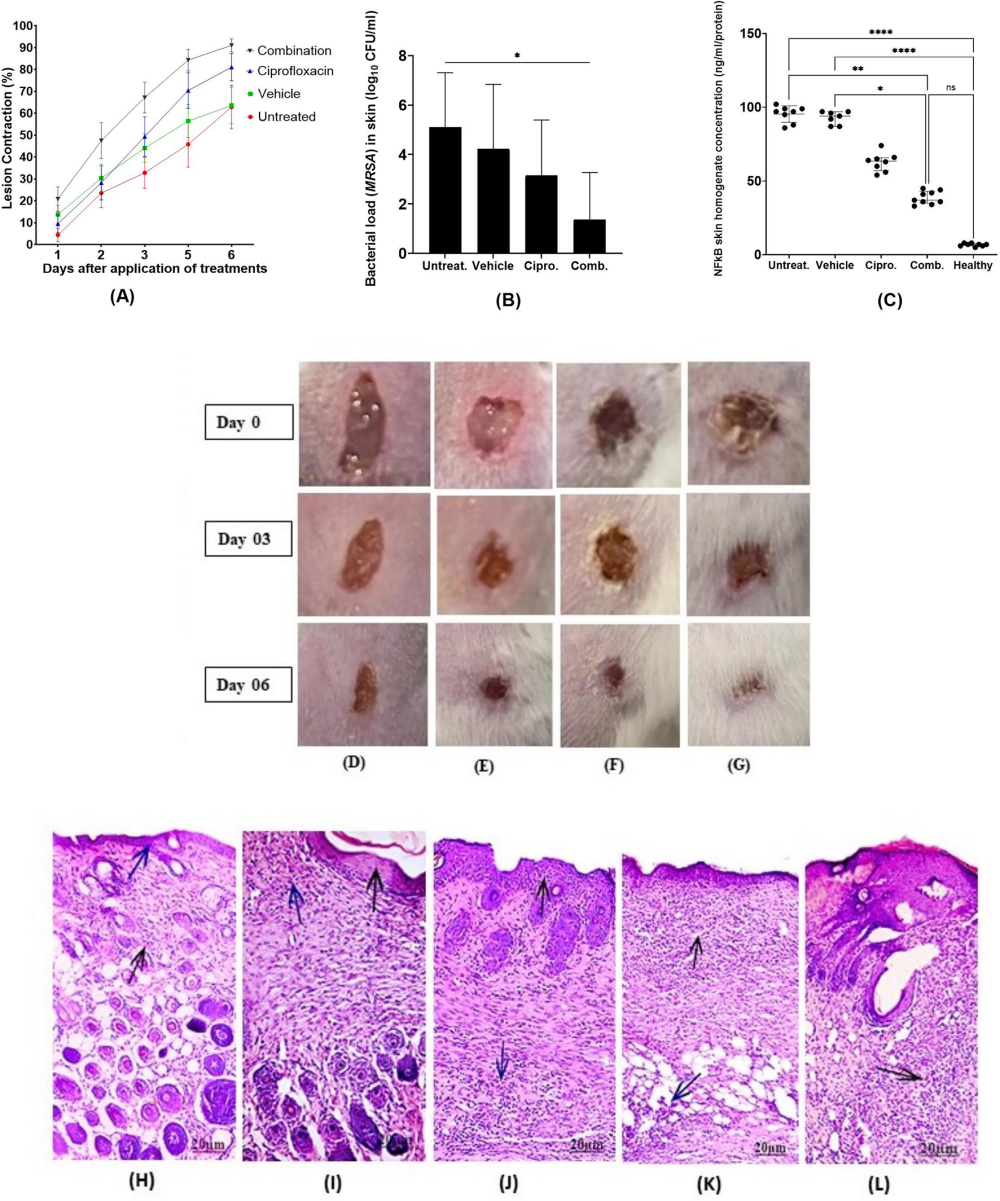
In vivo therapeutic efficacy in a diabetic murine MRSA skin infection lesion model*.* Diabetic mice (STZ 150 mg/Kg) were injected with MRSA N315 and after 4 days post-infection developed open lesions (Day 0). Animals were then treated topically once daily for 6 days with AgNPs-GTE loaded Cs-TPP-NPs combination hydrogel, ciprofloxacin hydrogel (positive control), blank vehicle hydrogel (negative control), or were left untreated. (**A**) Percentage of lesion contraction over 6 days of topical treatment with the AgNPs-GTE-loaded Cs-TPP-NPs combination hydrogel compared to ciprofloxacin (positive control), vehicle control, and untreated groups. Data are expressed as mean ± SD (n = 7 mice/group/experiment, n = 2 independent biological experiments). Statistical significance was determined by two-way ANOVA followed by Tukey’s multiple comparisons test (* *P* < 0.05, ** *P* < 0.01, *** *P* < 0.001, **** *P* < 0.0001; ns: not significant). (**B**) MRSA N315 bacterial load (CFU/g tissue) recovered from infection site on day 10 post-infection (day 6 post-treatment). Data are expressed as mean ± SD (n = 7 mice/group/experiment, n = 2 independent biological experiments). Statistical significance was determined using one-way ANOVA followed Tukey’s multiple comparisons test (*P < 0.05). (**C**) NF-κB expression levels in skin tissue across experimental groups including healthy controls. Data are expressed as mean ± SD (n = 7 mice/group/experiment, n = 2 independent biological experiments). Statistical significance was determined using one-way ANOVA followed by Kruskal–Wallis’s test (* *p* < 0.05, ** *p* < 0.01, **** *p* < 0.0001, ns non-statistically significant). (**D**–**G**) Representative images illustrating the diabetic MRSA skin infection lesion model showing rate of healing after treatment with (**D**) untreated, (**E**) vehicle-treated, (**F**) ciprofloxacin-treated, and (**G**) AgNPs-GTE-loaded Cs-TPP-NPs combination on days 0, 3, and 6. (**H**–**L**) H&E-stained skin tissue Sects. (200x). Healthy group (**H**) showed normal histological structure of epidermal (blue arrow) and dermal layers (black arrow). Combination-treated group (**I**) showed regeneration of epidermal layer (black arrow) and minimal inflammatory infiltration of dermal layer (blue arrow). Ciprofloxacin-treated group (**J**) showed regeneration of all epidermal layer (black arrow) with moderate inflammatory cells infiltration in the dermis (blue arrow). Untreated (**K**) and vehicle (**L**) groups showed massive polymorphonuclear cells infiltration with poorly organized granulation tissue (black arrow) and extended to subcutaneous layer (blue arrow) (H&E × 200). Statistical analysis was performed using GraphPad Prism software (version 10.3.0).

The lesion area was measured daily for 6 days. The AgNPs-GTE-loaded Cs-TPP-NPs hydrogel significantly enhanced lesion contraction rates compared to other treatments. By day 3, the combination hydrogel treated group showed a mean lesion contraction of 67%, outperforming ciprofloxacin hydrogel (49%), vehicle hydrogel (44%), and the untreated group (32.7%). By day 6, the contraction rates increased to 91% for the AgNPs-GTE-loaded Cs-TPP-NPs combination hydrogel, followed by ciprofloxacin hydrogel (81%), vehicle hydrogel (63.5%), and the untreated group (62.9%) (Fig. 9A). These findings suggested that the AgNPs –GTE loaded Cs-TPP-NPs combination hydrogel is responsible for accelerated healing rates at the initial stage of the healing process.

### AgNPs-GTE loaded Cs-TPP-NPs hydrogel showed reduction in bacterial load

The bacterial counts recovered from the skin lesion patches of the mice and the bacterial dissemination reflected by detecting the bacterial count recovered from the spleen were assessed at day 10 post infection (termination day). Mice treated with AgNPs-GTE-loaded Cs-TPP-NPs combination hydrogel showed a significant reduction (*p* < 0.05) in bacterial counts from skin lesions compared to the untreated (negative control) group (Fig. 9B). While for spleen bacterial counts, the results revealed that no bacterial dissemination was found in all MRSA injected groups.

### AgNPs-GTE loaded Cs-TPP-NPs hydrogel showed highest anti-inflammatory activity

NF-κB concentration in mice skin tissue of the five groups (untreated, vehicle, ciprofloxacin, AgNPs-GTE loaded Cs-TPP-NPs combination and healthy group) was determined using ELISA kit. The results showed no significant difference between the AgNPs-GTE-loaded Cs-TPP-NPs combination hydrogel treated group and the healthy group. However, a significant (*p* < 0.05) reduction in the NF-κB concentration was observed in the combination-treated group compared to the negative controls (untreated and vehicle). These results highlighted the anti-inflammatory activity of the AgNPs-GTE loaded Cs-TPP-NPs combination (Fig. 9C).

### Histopathological assessment of skin specimen

Histopathological examination revealed superior healing in the AgNPs-GTE loaded Cs-TPP-NPs combination hydrogel-treated group, which showed complete epidermal regeneration covered by thin eosinophilic layer of keratin (score 4), minimal inflammation (score 1), well-organized granulation tissue (score: 2), and regularly arranged mature collagen fibers (score 4) (Fig. 9I) and (Table 4). This was comparable to the ciprofloxacin hydrogel-treated group, though the latter exhibited moderate inflammation mainly macrophages and few numbers of neutrophils (score 2) and less organized collagen fibers (score: 3) (Fig. 9J) and (Table 4).

**Table 4:** Histological scale of wound healing.

Groups	Epithelization	Polymorphonuclear leucocytes	Fibroblasts	New vessels	Collagen
Healthy	Normal histological structure of skin layers without significant pathological changes				
Combination	4	1	2	1	4
Ciprofloxacin	4	2	2	2	3
Untreated	3	4	1	2	2
Vehicle	3	4	1	2	2

The untreated and vehicle-treated groups showed complete regeneration of the epidermal layers covered by very thin eosinophilic layer of keratin (score: 3). However, Massive inflammatory cells infiltration mainly polymorphonuclear cells and macrophages (score 4), disorganized collagen fibers (score 2), and minimal neo-angiogenesis (score: 1) was observed (Fig. 9K and L) and (Table 4). These findings highlighted the enhanced regenerative potential of AgNPs-GTE loaded Cs-TPP-NPs combination hydrogel compared to controls.

## Discussion

Non-healing diabetic wounds and foot skin infections in diabetic patients are a common, complex, and costly global public health issue^72^. In addition to producing significant morbidities, these infections account for the majority of diabetes-related hospital admissions and are the most prevalent proximal cause of amputations^73^. The excessive use of antibiotics in the treatment of skin infections has been linked to the development of antibiotic resistance. Consequently, to overcome this global health concern and to minimize the high cost of treatment, plant materials and a green synthesis of different nanoparticles has been studied as an alternative approach for diabetic skin infections.

In the current study, gamma irradiation of green tea demonstrated effective microbial decontamination, with a significant reduction in total microbial count at 5 kGy and complete elimination at 7 kGy. These results align with findings by Khawory et al., who reported that higher doses of gamma radiation effectively reduce microbial loads, including bacteria, fungi, and spores, in plant materials^74^. HPLC chromatographic analysis of GTE before and after irradiation revealed preservation of key phytochemical constituents. This observation is consistent with a previous study on 3 medicinal plants (*Euodia malayana*, *Gnetum gnemon*, and *Khaya senegalensis*), which showed unchanged chromatographic profiles post-irradiation. A slight increase in peak intensity was noted in irradiated samples, potentially due to the elimination of microbial contamination following gamma radiation^75^.

Eco-friendly green synthesis of nanoparticles is increasingly preferred over conventional physical and chemical methods due to its simplicity, low cost, and avoidance of hazardous reagents and toxic organic solvents. Traditional physicochemical approaches often involve harsh conditions and expensive, toxic chemicals, posing risks to human health and the environment. In response, biosynthesized nanoparticles have gained prominence, especially in biomedical application^76^. In this study, different nanoparticles were synthesized using GTE and gamma irradiation., where phytochemicals in GTE acted as both reducing and stabilizing agents, facilitating the bio-reduction of metal ions into nanoparticles^77^. Gamma irradiation, known for its high penetration and energy, produced free radicals in precursor solutions, further promoted the reduction of metal ions into high-purity nanoparticles^78^.

Characterization by transmission electron microscopy (TEM) and dynamic light scattering (DLS) confirmed the nanoscale size and distinct morphological features of the synthesized nanoparticles. The larger particle size obtained from DLS compared to TEM can be explained by the measurement principles of each technique. DLS determines the hydrodynamic diameter of nanoparticles in suspension, incorporating the solvation layer and potential aggregation effects in suspension, while TEM measures the actual core size of dried nanoparticles^79^. The negative average zeta potential results suggested that the capping biomolecules present on the biosynthesized NPs provide electrostatic repulsion between the synthesized NPs, contributing to nanoparticle stability^80^. FTIR spectrum further revealed that carboxylic and hydroxyl functional groups, were bound to the metal ions, potentially stabilizing the nanoparticles and preventing their aggregation by proteins or polysaccharides^43^.

Crucially, the successful synthesis and crystalline nature of AgNPs and ZnONPs were validated by XRD findings, which demonstrated similar types of peak indices for the crystalline nature of AgNPs and ZnONPs consistent with previous studies^81,82^. The sharp and well-defined peaks indicate high crystallinity and phase purity. This was further supported by the EDS profiles and elemental mapping, which confirmed the specific elemental signature of Ag and Zn demonstrated by their homogenous distribution within the nanoparticles matrices^83,84^. Collectively, these complementary analyses provide conclusive evidence of the physicochemical integrity of the synthesized materials.

The comparative evaluation of the tested formulations indicated that AgNPs represented the most potent antimicrobial and antibiofilm formula among the investigated formulations. Several previous studies have investigated nanoparticles based antimicrobial formulations similar to the current study, most reports demonstrated that AgNPs exhibited stronger antimicrobial activity than ZnONPs^85–87^. This apparent enhanced activity can be attributed to the distinctive physicochemical properties of silver nanoparticles, particularly their nanoscale size and high surface reactivity, which enhance interactions with bacterial cell wall and biofilm matrices^88^. Such interactions are known to disrupt membrane integrity, interfere with essential cellular functions, and limit biofilm formation. The observed efficacy of AgNPs is consistent with previous studies reporting their broad-spectrum activity against antibiotic-resistant strains, underscoring their potential as a strong antimicrobial agent^89–91^.

Plant based-conjugated silver nanoparticles (AgNPs) have been widely studied for antibacterial, antioxidant, anticancer, anti-inflammatory, antidiabetic, and anticoagulant properties. Bio-conjugation of Ag-based nanomaterials with plant extracts decreases their toxicity to biological systems and increases their therapeutic efficiency^92^. This study investigated the synergistic effects of combining GTE with different nanoparticles, aiming to assess the viability of such combinations for use in the healing purposes. Silver nanoparticles and GTE loaded Cs-TPP-NPs (1:1) combination demonstrated promising results showing the most potent synergistic antimicrobial activity against the four tested bacterial strains.

Several studies have explored the mechanisms underlying the modes of action of antimicrobial agents, many of which function by neutralizing bacterial surface charges, increasing membrane permeability, and ultimately reducing cell viability^93^. In this study, the mode of action of the most potent combination, AgNPs-GTE-loaded CS-TPP-NPs, was investigated against both Gram-negative (*E. coli*) and Gram-positive (MRSA) bacteria. The formulation exhibited strong bactericidal activity, primarily through disruption of the bacterial cell membrane, as evidenced by cytoplasmic leakage, increased potassium efflux, nucleic acid loss and inhibition of biofilm formation. The antibacterial mechanism of AgNPs typically begins with their adhesion and aggregation on the bacterial cell surface, followed by penetration and structural disruption of the cell membrane^94^, largely due to their nanoscale size and high surface area-to-volume ratio. AgNPs also enhance membrane permeability, induce reactive oxygen species, and interfere with DNA replication via silver ion release^95^. Additionally, chitosan’s positively charged ammonium groups facilitate electrostatic interactions with bacterial membranes, leading to membrane damage, leakage of intracellular components (DNA and RNA), and cell death, as reported by Li et al.^96^. Notably, MRSA showed greater susceptibility to AgNPs than *E. coli*, with higher levels of nucleic acid leakage, consistent with findings by Naik et al.^97^.

Hydrogels are 3D polymeric networks, that can swell and trap significant amounts of medicinal drugs and other compounds because of their porous structure. Hydrogels are 3D polymeric networks ideal for drug delivery due to their porous structure, controlled release profile, biocompatibility, and biodegradability^98^. Overall, the hydrogel network, with its increased porosity, is suitable material for wound dressing application to sustain a moist environment and promote breathability^99^ which are important for wound healing. Thus, hydrogels loaded with bioactive components have been widely explored for managing wound infections in diabetic models^17^. For example, a glucose-activated nanozyme hydrogel incorporating glucose oxidase and insulin within bimetallic Zn-Fe metal organic framework nanoparticles, demonstrated synergistic antibacterial activity, angiogenesis promotion, and accelerated wound closure in diabetic mice^100^. Similarly, a protein-based hydrogel composed of keratin, protocatechuic aldehyde, and zinc ions was developed as a drug carrier for the sustained release of phellopterin, a natural compound derived from the traditional Chinese medicinal plant *Angelica dahurica*. This hydrogel demonstrated significant antibacterial activity and promoted tissue regeneration in diabetic wound models^101^.

The in vitro studies, including MIC, MBC, time kill assays and fractional inhibitory concentration index confirmed that AgNPs-GTE loaded Cs-TPP-NPs combination exhibited the most potent antimicrobial activity. Therefore, it was formulated as a hydrogel formula and evaluated as a healing agent in a murine diabetic skin infection lesion model. The formulated hydrogel healing effect was demonstrated through the contraction in the skin lesions, the histological examination, and the reduction in NF-κB inflammatory mediators. Moreover, AgNPs-GTE loaded Cs-TPP-NPs combination significantly lowered the MRSA bacterial load. The in vivo murine model results confirmed the antimicrobial and anti-inflammatory activities of AgNPs and chitosan NPs along with GTE against MRSA. These results indicated that treating diabetic skin infections with the AgNPs-GTE loaded Cs-TPP-NPs combination hydrogel significantly improved the healing process by promoting cell proliferation, re-epithelization, collagen deposition, and angiogenesis. These results align with previous studies which reported that nano-silver dressings have the potential to aid in wound healing through their dual effects of antimicrobial and wound healing promotion^102^. The use of AgNPs in advanced wound dressings, hydrogels, and coatings has considerably improved wound care by preventing infections, accelerating tissue healing, and lowering problems associated with chronic wounds^103^. Additionally, previous studies^1,104–106^ reported similar results, suggesting that chitosan and GTE would accelerate wound healing by reducing excessive inflammation, and promoting proliferation, epithelialization, collagen deposition, and neovascularization, providing further evidence of the healing potential of green synthesized AgNPs, GTE and chitosan nanoparticles.

## Conclusions

AgNPs-GTE loaded Cs-TPP-NPs combination formulated as a hydrogel topical dosage form was found to accelerate the process of healing in diabetic mice and reduced the NF-κB and inflammatory mediators compared to ciprofloxacin. Additionally, the combined hydrogel significantly lowered the MRSA bacterial load. In conclusion, the in vivo mouse model results confirmed the antimicrobial and anti-inflammatory activities of AgNPs-GTE loaded Cs-TPP-NPs combination against MRSA.

## Supplementary Information

Supplementary Information.

## Data Availability

All data generated during this study are included in this published article and its supplementary information files.

## Abbreviations

AgNPs: Silver nanoparticles
AgNPs-GTE loaded Cs-TPP-NPs: Silver nanoparticles-green tea extract loaded chitosan-tripolyphosphate nanoparticles
AMR: Antimicrobial resistance
ARRIVE guidelines: Animal research reporting of in vivo experiments
AVMA: American veterinary medical association
CFU: Colony forming unit
Cs: Chitosan
Cs-TPP-NPs: Chitosan-tripolyphosphate nanoparticles
DLS: Dynamic light scattering
EDS: Energy-dispersive X-ray spectroscopy
EGCG: Epigallocatechin-3- gallate
EMB: Eosin methylene blue
FTIR: Fourier transform infrared spectroscopy
GT: Green tea
GTE: Green tea extract
GTI: Green tea infusion
HPLC: High-performance liquid chromatography
ICAM-1: Intercellular adhesion molecule-1
ICP-MS: Inductively coupled plasma mass spectrometry
IL-1: Interleukin-1
IL-6: Interleukin-6
IL-18: Interleukin-18
KBr Pellet: Potassium bromide pellet
kGy: Kilogray
MBC: Minimum bactericidal concentration
MDR: Multidrug-resistant
MHA: Mueller–Hinton agar
MIC: Minimum inhibitory concentration
MRSA: Methicillin-resistant *Staphylococcus aureus*
MSA: Mannitol salt agar
NA: Nutrient agar
NF-κB: Nuclear factor Kappa B
NMs: Nanomaterials
NPs: Nanoparticles
OD: Optical density
PBS: Phosphate buffer saline
PMNL: Polymorphonuclear leucocytes
REC: Research ethics committee
ROS: Reactive oxygen species
SDA: Sabouraud dextrose agar
SDB: Sabouraud dextrose broth
SEM: Scanning electron microscopy
SSA: Salmonella-shigella agar
STZ: Streptozotocin
TEM: Transmission electron microscopy
TM: Tube method
TSA: Tryptic soy agar
TSB: Tryptic soy broth
TNF-α: Tumor necrosis factor-alpha
UV–Visible: Ultraviolet–visible
VCAM-1: Vascular cell adhesion molecule-1
XRD: X-ray Diffraction
ZnONPs: Zinc oxide nanoparticles
γ (radiation): Gamma radiation
ΣFIC: Fractional inhibitory concentration index
