# Supplementary material for: Biosynthesis of ecofriendly antibacterial nanoparticles with healing effects in a murine diabetic skin infection model
Source: Sci Rep. 2026 Jun 15;16:18512. doi: 10.1038/s41598-026-54908-z (PMC13269799; doi:10.1038/s41598-026-54908-z)
Supplement: Supplementary file 1 — Supplementary Information. [file 41598_2026_54908_MOESM1_ESM.docx]

**Supplementary Figures**

**Biosynthesis of Ecofriendly Antibacterial Nanoparticles with Healing Effects in a Murine Diabetic Skin Infection Model**

Eman A. Mustafa^1,2^, Hanady G. Nada^2^, Walaa A. Eraqi ^3^, Rehab N. Shamma ^4^, Hala N. Elhifnawi^2^ and Nourtan F. Abdeltawab^3^

^1^Postgraduate Program in Microbiology and Immunology, Faculty of Pharmacy, Cairo University, Cairo, Egypt

^2^Drug Radiation Research Department, National Center for Radiation Research and Technology, Egyptian Atomic Energy Authority, Cairo, Egypt

^3^Department of Microbiology and Immunology, Faculty of Pharmacy, Cairo University, Cairo 11562, Egypt

^4^Department of Pharmaceutics and Industrial Pharmacy, Faculty of Pharmacy, Cairo University, Cairo 11562, Egypt

**
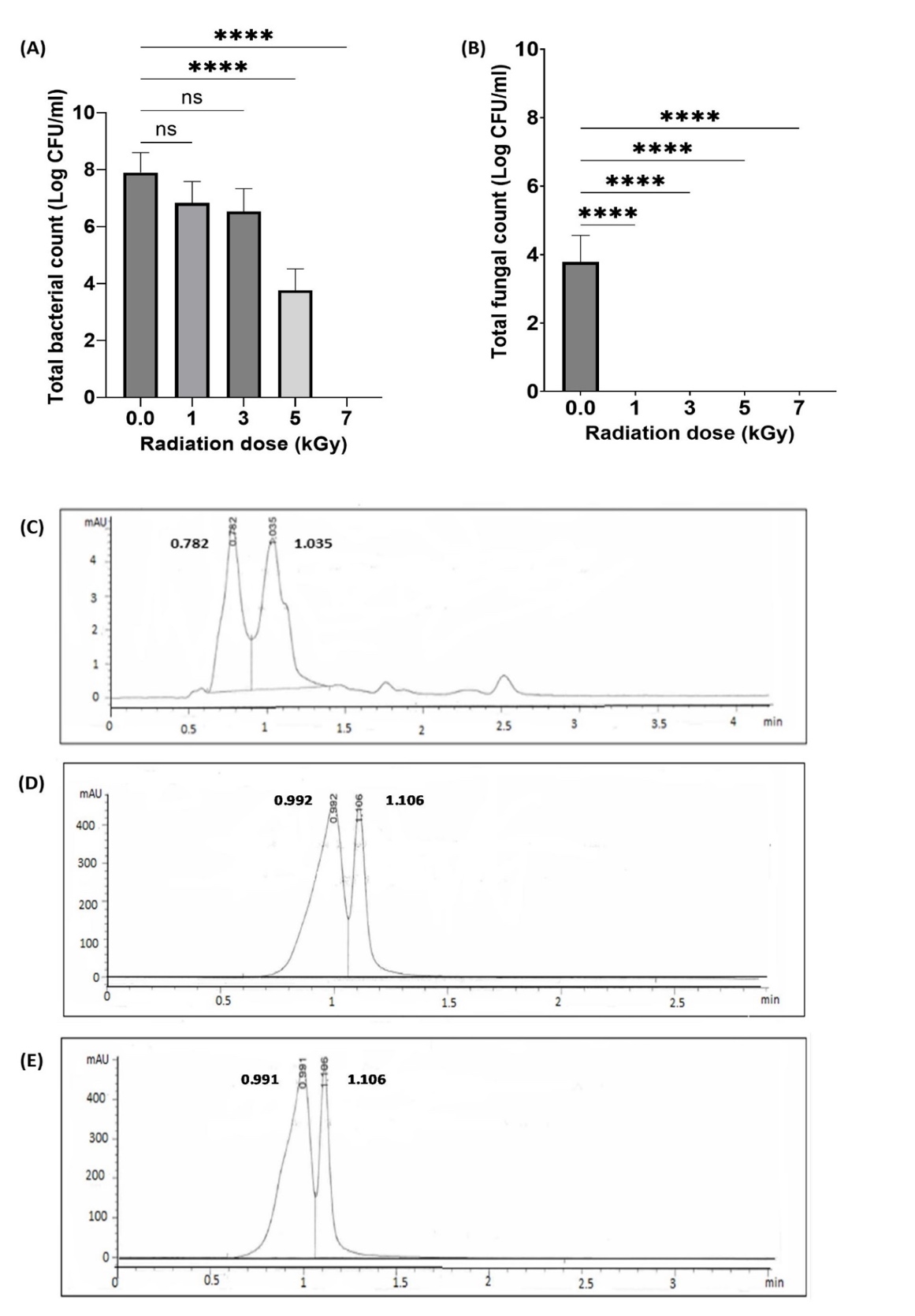
**

**Supplementary Figure 1. Effect of γ** **irradiation selected tested doses on bacterial and fungal load, and green tea components integrity. (A)** Total bacterial load (bacterial counts in colony forming unit/ ml (CFU/ml)) before and after exposure to different doses of γ irradiation. **(B)** Total fungal load (fungal count/ml) before and after exposure to different doses of γ irradiation. **(C)** HPLC chromatogram of standard EGCG. **(D)** HPLC chromatogram of green tea before irradiation. **(E)** HPLC chromatogram of green tea after 7 kGy irradiation selected dose. Data are represented as mean ± SD, n= 3, and statistical analysis was performed using one-way ANOVA followed by post hoc test (Tukey’s test) **** *p <* 0.0001, ns means non-significant. The statistical analysis was performed using GraphPad Prism software (version 10.3.0).


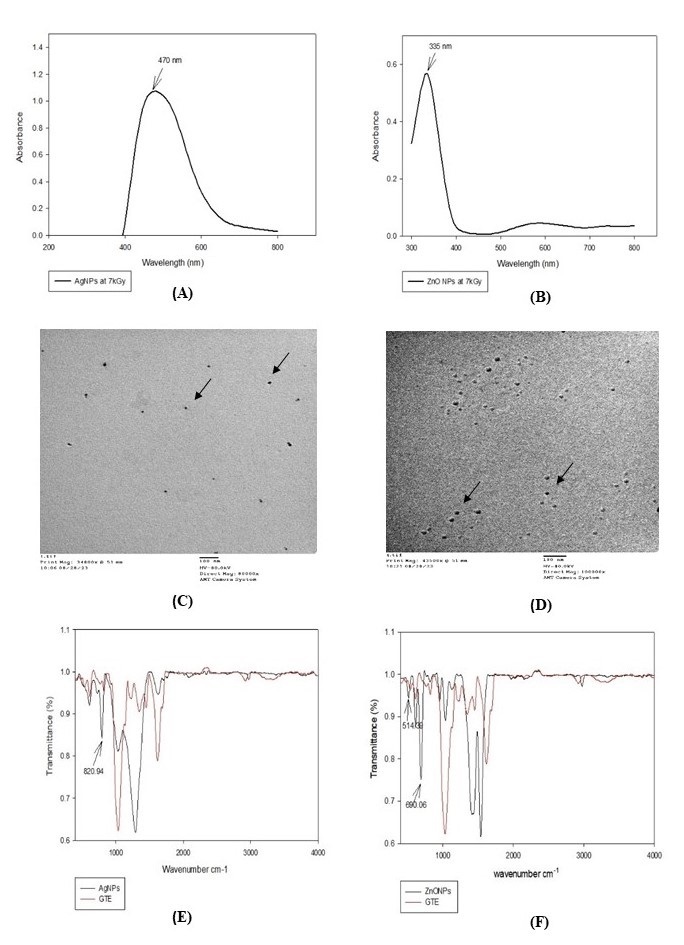


**Supplementary Figure 2. Multi-spectral and morphological characterization of synthesized metal nanoparticles.** UV-visible spectroscopy of **(A)** AgNPs and **(B)** ZnONPs. Transmission electron microscopy (TEM) images of synthesized **(C)** AgNPs and **(D)** ZnONPs. Fourier Transform Infrared (FTIR) spectra of **(E)** AgNPs and **(F)** ZnONPs in comparison to GTE alone.


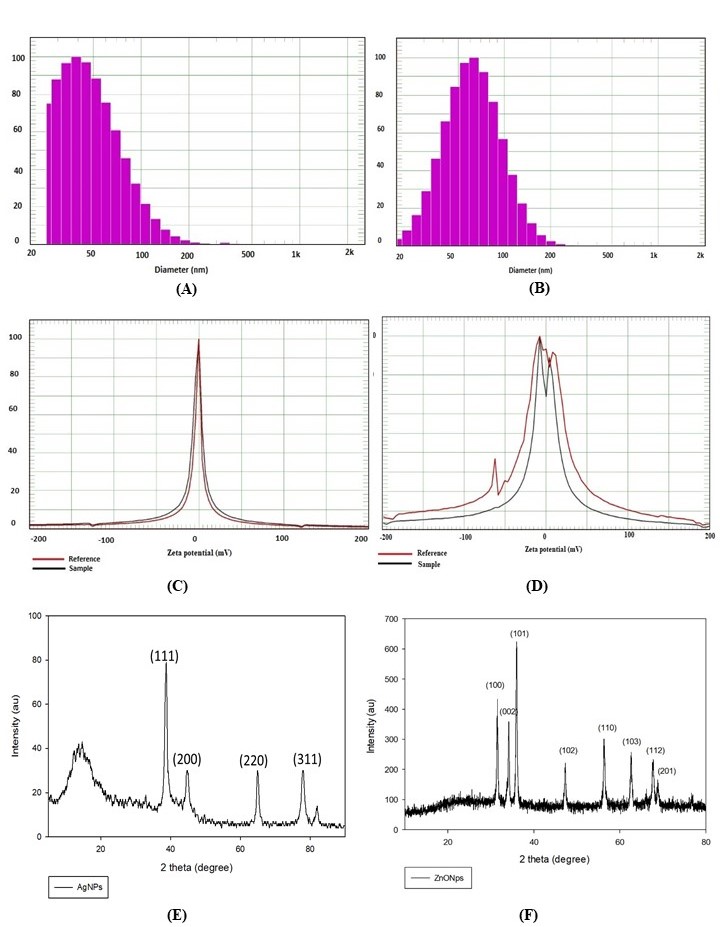


**Supplementary Figure 3. Size distribution and crystalline structure of synthesized metal nanoparticles.** Particle size distribution by dynamic light scattering (DLS) for **(A)** AgNPs and **(B)** ZnONPs. Zeta potential distribution of **(C)** AgNPs and **(D)** ZnONPs. X-ray diffraction (XRD) patterns spectrum confirming the crystalline nature of the synthesized **(E)** AgNPs and **(F)** ZnONPs.


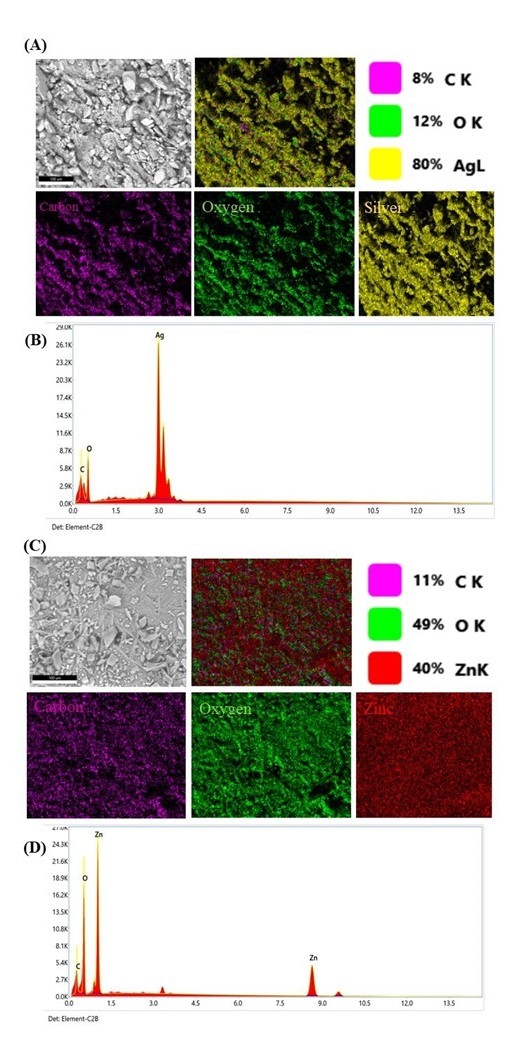


**Supplementary Figure 4. Elemental composition and surface analysis of synthesized metal nanoparticles. (A)** scanning electron microscopy (SEM)/energy dispersive X-ray spectroscopy (EDS) elemental mapping images of AgNPs **(B)** EDS spectrum confirming the presence of Ag in AgNPs **(C)** SEM/EDS elemental mapping images of ZnONPs **(D)** EDS spectrum confirming the presence of Zn in ZnONPs.


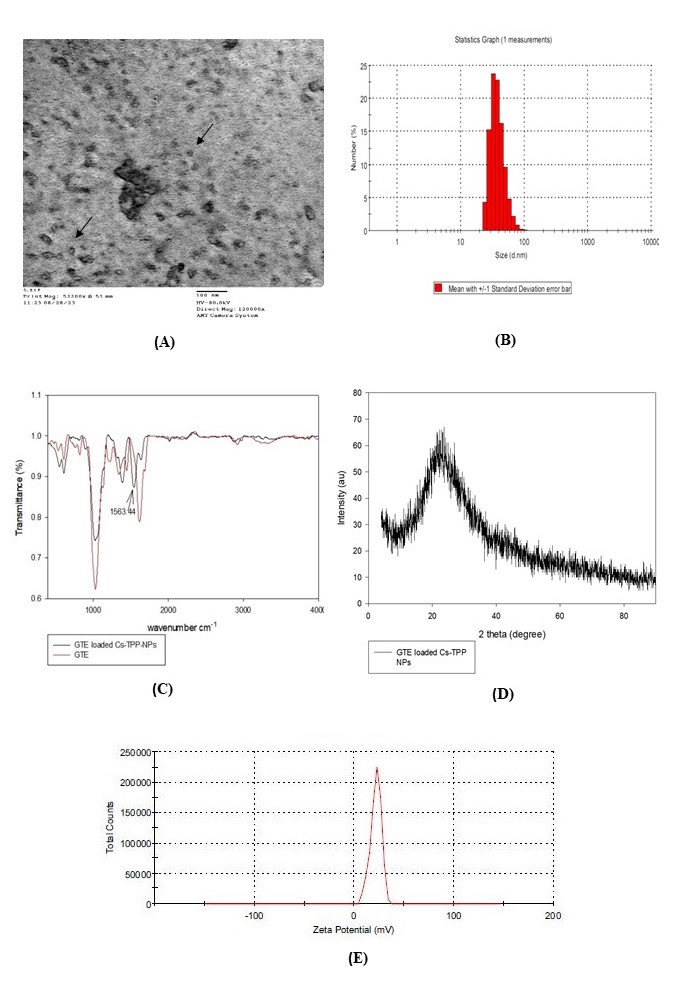


**Supplementary Figure 5. Characterization of synthesized GTE-loaded Cs-TPP-NPs. (A)** TEM morphology, (**B**) DLS size distribution, (**C**) FTIR spectra in comparison to GTE, (**D**) XRD pattern, and (**E**) Zeta potential of the GTE-loaded Cs-TPP-NPs.
